# A 32 kb Critical Region Excluding Y402H in *CFH* Mediates Risk for Age-Related Macular Degeneration

**DOI:** 10.1371/journal.pone.0025598

**Published:** 2011-10-12

**Authors:** Theru A. Sivakumaran, Robert P. Igo, Jeffrey M. Kidd, Andy Itsara, Laura J. Kopplin, Wei Chen, Stephanie A. Hagstrom, Neal S. Peachey, Peter J. Francis, Michael L. Klein, Emily Y. Chew, Vedam L. Ramprasad, Wan-Ting Tay, Paul Mitchell, Mark Seielstad, Dwight E. Stambolian, Albert O. Edwards, Kristine E. Lee, Dmitry V. Leontiev, Gyungah Jun, Yang Wang, Liping Tian, Feiyou Qiu, Alice K. Henning, Thomas LaFramboise, Parveen Sen, Manoharan Aarthi, Ronnie George, Rajiv Raman, Manmath Kumar Das, Lingam Vijaya, Govindasamy Kumaramanickavel, Tien Y. Wong, Anand Swaroop, Goncalo R. Abecasis, Ronald Klein, Barbara E. K. Klein, Deborah A. Nickerson, Evan E. Eichler, Sudha K. Iyengar

**Affiliations:** 1 Department of Epidemiology and Biostatistics, Case Western Reserve University, Cleveland, Ohio, United States of America; 2 Division of Human Genetics, Cincinnati Children's Hospital Medical Center, Cincinnati, Ohio, United States of America; 3 Department of Genetics, Case Western Reserve University, Cleveland, Ohio, United States of America; 4 Department of Ophthalmology, Case Western Reserve University, Cleveland, Ohio, United States of America; 5 Department of Genome Sciences, University of Washington, Seattle, Washington, United States of America; 6 Department of Biostatistics, Center for Statistical Genetics, University of Michigan School of Public Health, Ann Arbor, Michigan, United States of America; 7 Cole Eye Institute, Cleveland Clinic, Cleveland, Ohio, United States of America; 8 Department of Ophthalmology, Cleveland Clinic Lerner College of Medicine of Case Western Reserve University, Cleveland, Ohio, United States of America; 9 Research Service, Louis Stokes Cleveland Department of Veterans Affairs Medical Center, Cleveland, Ohio, United States of America; 10 Casey Eye Institute, Oregon Health & Science University, Portland, Oregon, United States of America; 11 Division of Epidemiology and Clinical Applications, National Eye Institute, Bethesda, Maryland, United States of America; 12 SNONGC Department of Genetics and Molecular Biology, Vision Research Foundation, Sankara Nethralaya, Chennai, India; 13 Singapore Eye Research Institute, Yong Loo Lin School of Medicine, National University of Singapore, Singapore, Singapore; 14 Centre for Vision Research, University of Sydney, Sydney, Australia; 15 Genome Institute of Singapore, Singapore, Singapore; 16 Departments of Ophthalmology and Genetics, University of Pennsylvania, Philadelphia, Pennsylvania, United States of America; 17 Institute of Molecular Biology, University of Oregon, Eugene, Oregon, United States of America; 18 Department of Ophthalmology and Visual Sciences, University of Wisconsin School of Medicine and Public Health, Madison, Wisconsin, United States of America; 19 Department of Medicine, Boston University, Boston, Massachusetts, United States of America; 20 Department of Ophthalmology, Boston University, Boston, Massachusetts, United States of America; 21 Department of Biostatistics, Boston University, Boston, Massachusetts, United States of America; 22 The EMMES Corporation, Rockville, Maryland, United States of America; 23 Department of Medical Retina, Vision Research Foundation, Sankara Nethralaya, Chennai, India; 24 Department of Glaucoma, Vision Research Foundation, Sankara Nethralaya, Chennai, India; 25 Centre for Eye Research Australia, University of Melbourne, East Melbourne, Australia; 26 Neurobiology Neurodegeneration and Repair Laboratory, National Eye Institute, Bethesda, Maryland, United States of America; 27 Kellogg Eye Center and Department of Human Genetics, University of Michigan, Ann Arbor, Michigan, United States of America; 28 Howard Hughes Medical Institute, University of Washington, Seattle, Washington, United States of America; University of Helsinki, Finland

## Abstract

Complement factor H shows very strong association with Age-related Macular Degeneration (AMD), and recent data suggest that multiple causal variants are associated with disease. To refine the location of the disease associated variants, we characterized in detail the structural variation at *CFH* and its paralogs, including two copy number polymorphisms (CNP), CNP147 and CNP148, and several rare deletions and duplications. Examination of 34 AMD-enriched extended families (N = 293) and AMD cases (White N = 4210 Indian = 134; Malay = 140) and controls (White N = 3229; Indian = 117; Malay = 2390) demonstrated that deletion CNP148 was protective against AMD, independent of SNPs at *CFH*. Regression analysis of seven common haplotypes showed three haplotypes, H1, H6 and H7, as conferring risk for AMD development. Being the most common haplotype H1 confers the greatest risk by increasing the odds of AMD by 2.75-fold (95% CI = [2.51, 3.01]; p = 8.31×10^−109^); Caucasian (H6) and Indian-specific (H7) recombinant haplotypes increase the odds of AMD by 1.85-fold (p = 3.52×10^−9^) and by 15.57-fold (P = 0.007), respectively. We identified a 32-kb region downstream of Y402H (rs1061170), shared by all three risk haplotypes, suggesting that this region may be critical for AMD development. Further analysis showed that two SNPs within the 32 kb block, rs1329428 and rs203687, optimally explain disease association. rs1329428 resides in 20 kb unique sequence block, but rs203687 resides in a 12 kb block that is 89% similar to a noncoding region contained in ΔCNP148. We conclude that causal variation in this region potentially encompasses both regulatory effects at single markers and copy number.

## Introduction

Age-related macular degeneration (AMD) is the leading cause of visual dysfunction and blindness in developed countries, and a rising cause in underdeveloped countries. In the United States (US), its prevalence in the population over age 65 years is 9% and increases to 28% in those over 75 years [1]. AMD is characterized by progressive degeneration of the macula, causing central field vision loss. A characteristic feature of AMD is the formation of deposits in the macula, called drusen, which may progress to either geographic atrophy or subretinal neovascularization, manifestations of late AMD. Several genetic and environmental risk factors influence disease susceptibility [2], [3]. Genes involved in the complement pathway have especially been implicated in disease pathogenesis [4], [5], [6], [7], [8], [9], [10], [11], [12], [13].

A major locus for AMD pathogenesis, *CFH*, was identified through linkage and Genome-wide association studies (GWAS) [4], [8], [9]. A nonsynonymous single nucleotide polymorphism (SNP) in *CFH*, rs1061170 (also known as Y402H), was proposed as the major risk variant with odds ratios ranging from 1.4 to 7.4 (reviewed in Patel et al. [14]). In the contiguous region, a common deletion involving *CFHR3* and *CFHR1* was also shown to confer protection against AMD [15]. However, deeper analysis of the data led to *CFH* intronic SNPs being more significantly associated with AMD than rs1061170 [16], [17], suggesting that other unidentified variants play an important role in disease pathogenesis. We therefore systematically examined SNPs and structural variation in the *CFH* region and propose a unifying hypothesis for AMD risk.

## Results

### Structural Variation at the RCA Gene Cluster

**Figure 1:**
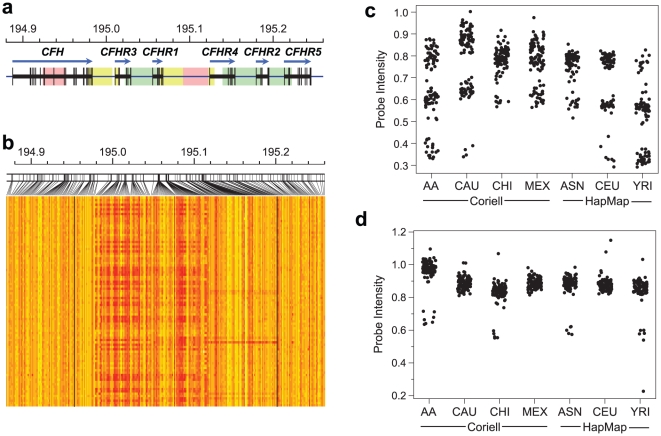
Detection of CNPs in the reference samples. (**1a**) Schematic diagram showing the location of the genes and segmental duplications in the RCA gene cluster. Distal areas that are highly similar to one another are represented using the same color. (**1b**) Heat map showing normalized probe intensities in 90 HapMap Yoruban (YRI) samples. Yellow indicates the high probe intensity and red indicates low intensity; clustering of low intensity (red) occurs in regions with CNP 147 and CNP 148. Black vertical lines denote the boundaries of the region that incorporates CNP147 and CNP148. (**1c**) and (**1d**) Genotypes of CNP147 and CNP178, respectively, across different reference groups. y axis is probe intensity and x axis is reference groups (see Table S3 for CNP genotype frequencies); AA: Coriell African American, CAU: Coriell Caucasian, CHI: Coriell Chinese, MEX: Coriell Mexicans, ASN: HapMap Asian, CEU: HapMap Caucasian, YRI: HapMap Yorubans.

**Figure 2:**
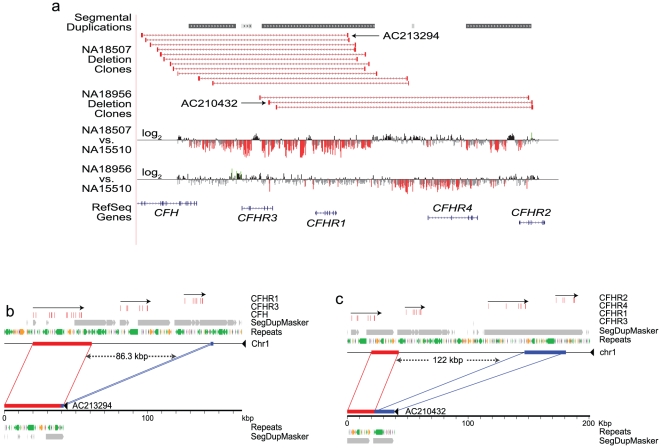
Validation of CNPs in the HapMap samples. (**2a**) UCSC browser view (http://humanparalogy.gs.washington.edu/) showing organization and structural variation in the RCA gene cluster. Red bars indicate the sites of structural variation in eight individuals underwent fosmid pair-end sequencing. Deletions at CNP147 and CNP148 were using high-resolution tiling-path custom array-based CGH. Probes with log2 ratios below or above a threshold of 1.5 s.d. from the normalized mean log2 ratio are colored red (deletions) or green (duplication), respectively. (**2b**) and (**2c**) Alignment of sequenced fosmid inserts against the human genome assembly (build36) confirms the extent of two deletions in the CFHR cluster on chromosome 1. Clone AC213924, derived from sample NA18507, corresponds to deletion CNP147 (**2b**). This variant removes 86.3 kbp of sequence (chr1:194,988,828–195,075,129, build36), resulting in loss of *CFHR3* and *CFHR1* genes. Clone AC210432, derived from sample NA18956, corresponds to deletion CNP148 (**2c**). This variant removes 122.0 kbp of sequence (chr1:195,049,336–195,171,294), resulting in loss of *CFHR1* and *CFHR4* genes. Red boxes: RefSeq transcripts, with orientation indicated by arrows. Grey boxes: regions of segmental duplication as predicted by SegDup Masker. Common repeats were identified using Repeat Masker. Purple: SINEs, Green: LINEs, Pink: DNA elements, Light Grey: Low Complexity sequence, Black: Simple Repeats.

Our initial examination of the genomic sequence between *CFH* and *CFHR5* revealed duplicated segments, including several novel, highly similar, homologous regions (Figure S1, Text S1 and Table S1). In general, the high sequence identity among duplicated regions increases the probability of structural rearrangements due to non-allelic homologous recombination. None of the previous studies thoroughly screened the RCA gene cluster for structural variation, or considered copy number information and SNPs together to identify causal variants at *CFH*. Therefore, we conducted a detailed survey of the structural variation in 1582 reference samples. This revealed existence of two CNPs, CNP147 and CNP148, in addition to several rare copy number variations (CNVs) in the RCA gene cluster (Figure 1a–d and Table S2). Both CNPs most often carry deletions rather than duplications, and the frequencies of these deletions vary among different reference populations (Table S3). We validated these CNPs in the reference samples using fosmid pair-end sequencing, array comparative genomic hybridization (CGH), depth of coverage analysis of genomic sequence, and a PCR based deletion screening strategy (Figure 2a, Figures S2 and S3). Finally, we sequenced the two deletion variants, after subcloning them in fosmid clones [18] (Figure 2b, 2c), which provided single basepair resolution for each deletion. The deletion at CNP147 (ΔCNP147) was about 86.3 kb (chr1: 194,988,828–195,075,129, National Center for Biotechnology Information (NCBI) build 36), although it was earlier reported to be about 84.6 kb [15], and included *CFHR3* and *CFHR1*. The distal deletion at CNP148 (ΔCNP148) spanned 122.0 kb, encompassing *CFHR1* and *CFHR4* (chr1:195,049,336–195,171,294, NCBI build 36).

**Figure 3:**
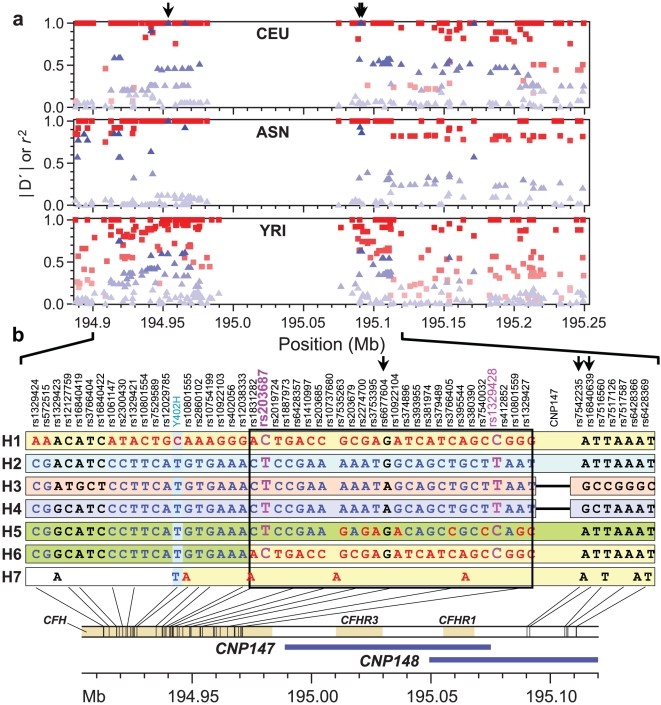
Haplotypes and linkage disequilibrium (LD) in the region surrounding CNP147. (**a**) Distribution of r^2^ and D′ values for SNPs with CNP147 in three HapMap populations. The squares represent the D′ values while the triangles indicate the r^2^ values. Different shades of squares and triangles indicate weak (light shade) to strong LD (dark shade). ΔCNP147 shows perfect LD with nearby SNPs in non-Africans. The arrows indicate the SNPs used as a proxy to predict ΔCNP147 in our AMD custom Illumina panel. (**b**) Haplotypes in the *CFH* region observed in clinical cohorts. Haplotypes were constructed from genotypes of 70 SNPs located in the *CFH* region using PHASE2.1.1. Location and alleles of key selected SNPs are given here for expediency. The risk-associated allele at each locus is shown in red and the protective alleles at the corresponding locus are in blue. The bottom panel shows the location of CNP147 and CNP148. ΔCNP147 is indicated by a thin black line in haplotypes H3 and H4. Except H3 and H4, all haplotypes carry normal constitution at CNP147. The critical region, wherein the putative AMD susceptibility variant lies, is enclosed in black box for clarity and the two best SNPs rs203687 and rs1329428 are highlighted in pink. H1 and H6 are risk bearing, and H2–H4 are protective in nature. The role of H5, which carries risk alleles at several loci in block2 in causing disease is not clear. The Indian cohort has a rare risk recombinant haplotype H7, with non-risk allele T at rs1061170 present in 4% of the chromosomes. The most common risk haplotype among the Indians was equivalent to H1 as seen in Caucasians.

We further determined the ancestral origin of these CNPs using phylogenetic and linkage disequilibrium (LD) analysis, which demonstrated that these common deletions were recurrent in origin in Africans, although the results showed a single ancestral origin of ΔCNP147 in Caucasians and Asians (Figure 3a and Figure S4). Since ΔCNP147 in Caucasians resides on an ancestral haplotype, we were able to find perfect correlation (r^2^ = 1) between ΔCNP147 and SNPs in the surrounding region, i.e., rs6677604, rs12144939, rs7542235, rs16840639 and rs2996127. Though perfect LD between rs6677604 and ΔCNP147 has been reported before [15], [19], [20], we found the exact extended haplotypic background on which the deletion occurred. We did not observe LD between ΔCNP148 and any nearby SNPs.

### A 32 kb Region in *CFH* is Important for AMD Risk

We sought to determine the causal variant(s) in clinical samples including 34 extended families and a non-familial dataset consisting of 4484 cases and 5736 controls from three different populations. We first determined the copy number at CNP147 and CNP148 using microarray intensity data. Direct assessment of copy numbers at CNP147 was not possible in the clinical samples, except in the Familial Age-related Maculopathy Study (FARMS) cohort, due to low probe coverage in many of the commercial arrays in this region. We, therefore, used tag SNPs to predict the deletions at CNP147. Predicted homozygous deletions were validated with PCR-based deletion screening (Figures S5 and S6). We constructed the haplotypes using SNP and CNP147 genotype data. We did not include CNP148 in the haplotype analysis because it is rare and occurred on multiple haplotypic backgrounds.

Haplotype analyses revealed six different common haplotypes (defined as present at a frequency of >1% in the samples) in the Caucasian datasets (Figure 3b). Using the H2 haplotype as baseline because it carried neither ΔCNP147 nor the C risk allele at codon 402 of *CFH*, we assessed the effect of all other haplotypes (Table 1, 2 and Table S4). The results showed that the most common H1 haplotype conferred the greatest risk relative to H2 by increasing the odds of AMD by 2.75-fold (95% CI = [2.51, 3.01], p = 8.31×10^−109^) in the Caucasian non-family cohorts. Haplotypes H3 and H4 with ΔCNP147 were found to confer protection against AMD over and above the base H2 haplotype (OR = 0.74 p = 1.83×10^−5^; and OR = 0.70, p = 2.81×10^−4^), demonstrating that the deletions are indeed protective. H3 and H4 are virtually identical to H2 with the exception of the presence of ΔCNP147; phylogenetic analysis suggests that ΔCNP147 may have originated on the H2 background (Figure S4).

**Table 1:** Association of CFH haplotypes in the FARMS cohort.

		Haplotype Association	
Haplotype	Frequency	Effect	LRT p-value
H1	0.5189	0.657	9.46×10^−05^
H2	0.1065	Base	-
H3	0.067	−0.279	0.272
H4	0.0206	−0.293	0.485
H5	0.1615	−0.008	0.714
H6	0.0653	0.467	0.082

**Table 2:** Association of CFH haplotypes with AMD in different clinical cohorts.

		Haplotype Frequency			Haplotype Association@		
Cohort	Haplotype	Cases	Controls	Total	OR	95% CI	p-value
Custom Illumina Panel*	H1	0.5152	0.3263	0.4210$	2.25	1.96, 2.58	1.35×10^−30^
	H2	0.1061	0.1916	0.1488$	Base	-	-
	H3	0.0611	0.1205	0.0908$	0.70	0.56, 0.87	1.15×10^−03^
	H4	0.0285	0.0624	0.0454$	0.61	0.46, 0.82	8.49×10^−04^
	H5	0.1459	0.1416	0.1438$	1.46	1.21, 1.75	6.93×10^−05^
	H6	0.0379	0.0322	0.0351$	1.83	1.33, 2.54	2.42×10^−04^
MPM Panel#	H1	0.5802	0.3317	0.4878	3.35	2.91, 3.86	1.73×10^−62^
	H2	0.0888	0.2000	0.131	Base	-	-
	H3	0.0593	0.1230	0.0832	0.89	0.73, 1.10	0.275
	H4	0.0209	0.0548	0.0336	0.80	0.59, 1.08	0.143
	H5	0.1435	0.1570	0.1477	1.73	1.45, 2.06	6.24×10^−10^
	H6	0.0359	0.0322	0.0353	2.19	1.61, 2.99	2.42×10^−07^
AREDS	H1	0.5855	0.3193	0.499	2.45	1.76, 3.41	9.70×10^−08^
	H2	0.0768	0.1566	0.1027	Base	-	-
	H3	0.0536	0.1687	0.091	0.43	0.26, 0.71	0.001
	H4	0.0130	0.0392	0.0215	0.48	0.19, 1.22	0.120
	H5	0.1174	0.1386	0.1243	1.06	0.65, 1.719	0.830
	H6	0.0333	0.0211	0.0294	2.27	0.89, 5.79	0.087
Indian	H1	0.4291	0.2391	0.3414	2.05	1.27, 3.30	0.003
	H2	0.0597	0.1696	0.1104	Base	-	-
	H3	0.0746	0.1391	0.1044	0.63	0.31, 1.26	0.190
	H4	0.0858	0.1870	0.1325	0.54	0.28, 1.07	0.078
	H5	0.0858	0.1217	0.1024	0.59	0.29, 1.22	0.150
	H7	0.0672	0.0043	0.0382	15.57	2.12, 114.30	0.007
SiMES	H1	0.0786	0.0768	0.0767	0.99	0.62, 1.57	0.950
	H2	0.4286	0.4161	0.4176	Base	-	-
	H3	0.0143	0.0100	0.0102	1.69	0.58, 4.90	0.340
	H4	0.0357	0.0393	0.0389	0.92	0.47, 1.80	0.800
	H5	0.3893	0.4013	0.4003	0.99	0.76, 1.29	0.950
Meta CC^1^	H1				2.75	2.51, 3.01	8.31×10^−109^
	H2				Base	-	-
	H3				0.74	0.65, 0.85	1.83×10^−05^
	H4				0.70	0.58, 0.85	2.81×10^−04^
	H5				1.54	1.37, 1.73	1.73×10^−13^
	H6				1.85	1.51, 2.27	3.52×10^−09^
Meta All^2^	H1						2.00×10^−90^
	H2						Base
	H3						8.07×10^−05^
	H4						0.003
	H5						1.44×10^−10^
	H6						4.52×10^−09^

*Odds ratio and p-values obtained from meta-analysis is given here and the results from the individual cohorts are seen in the Table S4.

$ Frequency from the pooled data (all cohorts).

# Results from the pooled data.

@ Association using H2 haplotype as baseline, adjusted for covariates described in Table S8.

1 Meta analysis of all Caucasians non-familial cohorts.

2 Meta analysis of all Caucasian cohorts (non-family cohorts and family cohort in Table 2) and p-value indicates the Fisher p-value.

We also identified a recombinant haplotype (H6), with a frequency of ∼4%, which conferred risk for AMD (OR = 1.85; p = 3.52×10^−9^). Since this recombinant haplotype excludes most of the LD block 1 that carries codon 402, it was crucial in reducing the AMD-associated interval to a 32-kb block, not including rs1061170. We also found another haplotype, H5, without the C allele at rs1061170, that confers risk for AMD (OR 1.54; p = 1.73×10^−13^) over the H2 haplotype; however, the frequency of H5 haplotype is more in controls than cases. Analysis of each haplotype independent of H2 as baseline showed H5 a neutral haplotype, with OR not significantly different from 1 (data not shown). Unlike H1 or H6, which carried the same core 32-kb haplotype between markers rs9970784 and rs70620, H5 showed some differences but also carried many of the same risk alleles in the 32-kb core. Thus, it is not clear whether H5 is causal or neutral in the general population; additional studies are needed to confirm its role in the disease pathogenesis. The final support for the 32-kb critical region came from the Indian cohort, where another uncommon, possible recombinant, haplotype (H7) conferred risk for AMD (OR = 15.57; p = 0.007). In this case, a recombination event occurred between rs1061170 and rs10801555, but the rest of the haplotype was identical to the 32 kb core risk haplotype present in H1 and H6.

### rs1329428 and rs203687 are the best SNPs of AMD-association at *CFH*

**Figure 4:**
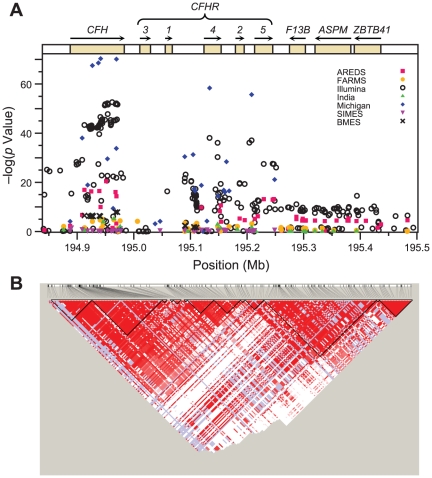
The strength of the association signal, as indicated by −log(p value) on the Y-axis, is plotted against SNP location on chromosome 1 on the X-axis. The (A) panel shows the p-values from individual clinical cohorts which are labeled in different colors and shapes (see Labels). The SNPs located within the 32 kb critical region show the best association signal in the 6 clinical datasets. Panel (B) shows the distribution of pair-wise linkage disequilibrium (D′) values between *CFH* and *ZBTB41*. The LD blocks are marked with black triangles; red indicates D′ values of 1 (complete LD) while white indicates D′ values of 0 (no LD whatsoever).

Traditional single SNP-disease association analyses showed consistently strong signals, which created an unusually wide footprint, from *CFH* to *ZBTB41* (Figure 4 and Table S5). This observation is potentially due to suppression of recombination as a result of structural variation between the RCA gene cluster haplotypes. In agreement with our haplotype results, the majority of SNPs in the 32 kb critical region showed very strong association with AMD (10^−45^<p<10^−133^). Li et al. [16] and Maller et al. [17] first showed that several SNPs in this region were more strongly associated with AMD than rs1061170; among the best ranked variants in these earlier reports were rs2274700 and rs1410996, respectively.

To select a minimal set of SNPs that explain the effect of *CFH* on AMD, we used forward selection while restricting our analysis to the 32 kb region for which all risk haplotypes have support. We identified rs1329428 and rs203687 as the markers showing best evidence for association with AMD. These SNPs entered the models with p-values = 2.6×10^−105^, and 8.7×10^−14^, respectively. After this, the model was saturated and no other SNPs entered the model. Both these variants are located in non-coding intronic regions (intron 15 and 9, with r^2^ = 0.553, respectively) and are in moderate LD with rs1061170 (r^2^ = 0.315 and 0.574, respectively). These variants have been shown to bind to transcriptional regulatory proteins (Figure S7). Bioinformatic analyses predict that the variant allele “T” at rs203687 abolishes binding sites for RE-BP1 and CBP100 and creates two new sites, for NF-1 and Oct-1. However, analysis of HapMap gene expression data showed no significant changes in expression of *CFH* (p>0.05) with change in the variant alleles at rs1329428 and rs203687. Evaluation of these SNPs in the retinal cells is needed to gauge the importance of these sites in the regulation of the *CFH* gene expression. It is also possible that these variants are in LD with the actual causal variant. Exploring this 32 kb region using next-generation resequencing and expression tools would be helpful in understanding the causal variants at this locus.

### ΔCNP148 is Independently Associated with AMD

We sought to determine whether the common deletions at CNP147 and CNP148 confer protection independent of SNPs at *CFH*, and conducted logistic regression with and without rs1329428 and rs203687 as covariates. Without inclusion of the most signficiant SNPs, an increase in the copy number at CNP147 shows a trend towards the risk. After inclusion of these markers, the p-value at ΔCNP147 did not reach significance (p>0.05) (Tables 3, 4 and S6). These results suggest that the effect at ΔCNP147 is not independent of the markers at *CFH*. In supporting this, Raychaudhuri et al. [21] and Fritsche et al. [22] showed marginal significance of AMD with ΔCNP147 after adjusting for key SNPs such as rs1061170 and rs10737680 or rs2274700 (p = 0.02 and 0.044, respectively). One possible explanation for this trend may be due to occurrence of ΔCNP147 on the H2 background, and the modest LD between CNP147 and rs1329428 and rs203687 (r^2^ = 0.462 and 0.261, respectively). Additional studies of ΔCNP147 occurring on other haplotypes are needed to confirm these results.

**Table 3:** Association of ΔCNP147 with AMD, with and without inclusion of rs1329428 and rs203687, as covariates.

	ΔCNP147 Frequency		ΔCNP147 association with AMD$			ΔCNP147 association with AMD@		
Cohorts	Cases	Controls	OR	95% CI	p-value	OR	95% CI	p-value
Custom Illumina^1^ – Meta-analysis	0.1052#	0.2119#	2.21	1.90, 2.58	4.50×10^−24^	1.12	0.92, 1.37	0.24
MPM Panel^2^	0.0948	0.2074	2.47	2.11, 2.88	2.77×10^−30^	1.04	0.85, 1.27	0.72
AREDS^3^	0.0855	0.2314	3.23	2.22, 4.72	1.89×10^−9^	1.41	0.87, 2.29	0.16
Meta-analysis*	—	—	2.34	2.10, 2.61	1.33×10^−52^	1.10	0.96, 1.26	0.15
Indian^4^	0.2129	0.3675	2.06	1.35, 3.14	7.74×10^−4^	NA	NA	NA
SiMES^2^	0.0504	0.0649	1.47	0.53, 4.08	0.46	NA	NA	NA

# Frequencies obtained from the pooled data.

$ After adjusting for significant covariates as described in Table S8.

@ After adjusting for rs1329428 and rs203687, in addition to significant covariates as described in Table S8.

*Meta-analysis of the Custom illumina panel, MPM panel and AREDS cohort.

1 ΔCNP147 predicted by A-G-C haplotype at rs6677604, rs7542235 and rs16840639.

2 ΔCNP147 predicted by “A” allele at rs6677604.

3 Since none of the ΔCNP147-specific SNPs are genotyped in AREDS,the “A” allele at rs2019727 that is highly correlated (r^2^ = 0.9) with this deletion in the Caucasians was used as proxy.

4 ΔCNP147 predicted by the “G” allele at rs7542235.

NA - not adjusted for rs1329428 and rs203687 as their genotypes are not available.

**Table 4:** CNP association in the FARMS cohort.

	Association of CNPs with AMD$			Association of CNPs with AMD@		
CNP	Effect	SE	LRT p-value	Effect	SE	LRT p-value
CNP147*	0.689	0.182	0.0002	0.42	0.23	0.07
CNP148*	1.260	0.520	0.016	1.110	0.310	0.03

*Increase in every copy at of the CNP with increase in the AMD score.

$ Association of CNPs after adjusting for age and age^2^.

@ Association of CNPs after adjusting for age, age^2^, rs1329428, and rs203687.

Interestingly, the presence of additional copies of CNP148 conferred risk for AMD after even adjusting for the effect of the two best SNPs at *CFH* (OR 2.24; p = 0.0023 and ß = 1.11; and p = 0.03, respectively in the custom Illumina panel and the FARMS samples), suggesting that ΔCNP148 confers protection against AMD independent of SNPs at *CFH*.

## Discussion

We systematically examined the *CFH* region to categorize variants responsible for AMD pathogenesis using multiple datasets from around the world. We first characterized structural variations and SNPs in the RCA gene cluster, and then assessed their effect on AMD pathogenesis. We identified two common deletions: ΔCNP147, which removes all of *CFHR3* and *CFHR1*, and ΔCNP148, which removes *CFHR1* and *CFHR4* in addition to a large segment of flanking non-coding sequence. Our results show that ΔCNP148, a rarer deletion that occurs on variety of haplotype backgrounds, appears to confer protection against AMD in addition to SNPs at the *CFH* locus. The protection conferred by this deletion may be due to reduction or absence of the *CFH*-related protein products (*CFHR1* or *CFHR4*), or alternatively, regulatory sequence in the deleted region may have a significant impact on disease pathogenesis. The C-terminal regions of *CFHR3*, *CFHR1* and *CFHR4* exhibit considerable homology with *CFH* SCRs 18–20, enabling them to compete with *CFH* for binding to C3b [23], In addition, *CFHR1* also competes with *CFH* for cell surface attachment in the process of inhibition of C5 convertase and terminal complex formation [24]. This leads to a reduction in inhibition of C3 convertase and anti-inflammatory activity, which results in host cell damage [24], [25]. In the absence of *CFHR1 (CFHR4)*, local *CFH* binding and activity is increased resulting in enhanced iC3b deposition and likely phagocytosis of opsonized particles, which may be advantageous for the clearance of cellular debris in the retina [21].

The area between *CFHR1*-*CFHR4* seems particularly vulnerable to recurrent rearrangements when evolutionary history is examined in conjunction with human-specific comparisons. Comparative primate genome analysis shows that chimpanzees have more extensive duplication architecture at the RCA locus than humans. Targeted array CGH of the region confirms increase of *CFHR* copies in the chimpanzee and the bonobo when compared to human, but the orangutan has reduced copy-number (Figure S8). Interestingly, a portion of *CFHR4* seems to have hyper-expanded in the great apes (bonobo and orangutan). Drusen formation, a precursor to sight-threatening late AMD, is polymorphic in the Old World monkey, the rhesus macaque [26]. Macaques diverged from the common ancestor to humans approximately 25 million years ago, and the shared phenotypic features of retinal drusen formation in humans and macaques suggest that the mechanism is old and evolutionarily preserved. As described in the results, the core sequence containing AMD disease susceptibility variants shows significant homology with *CFHR1*-*CFHR4*. These duplicated tracks will need to be examined in greater molecular detail to characterize the exact causal mechanism.

Within human samples, we observed over 25 rare structural variant events in the RCA gene cluster in a sample size of 1875 individuals (1582 reference and 293 clinical) using high resolution SNP arrays (Tables S2 and S7). The majority of these events are smaller than 25 kb in size. About half of them are losses, affecting intergenic regions of *CFH*-related genes. Four of the rare events found in FARMS are larger than 25 kb. Two are deletions observed in a single individual. Since these events were *de novo* in nature, we were unable to predict whether they occurred on the same chromosome or on opposite homologs based on the information from the arrays alone (Table S7). The smaller of these two deletions results in the loss of the region encompassing exons 10–16 of *CFH*. Although we cannot be certain, this effect is likely to increase susceptibility to AMD because the core variant(s) causing disease lies within the boundaries of this deletion. The larger deletion of 209 kb removes one of the copies of *CFHR1*, *CFHR4*, *CFHR2* and part of *CFHR5*. The effect of the latter deletion is likely to be protective, but its impact is currently unknown because the deletion extends far beyond ΔCNP148. The participant carrying both deletions is only 45 years old, when incipient disease is not yet apparent. This individual has an average score of 2 on the Wisconsin age-related maculopathy grading system (WARMGS) score, which is approximately equal to having retinal hard distinct drusen <63 µm in diameter [27]. Supporting the hypothesis of recurrent rearrangements, a rare, large deletion, involving *CFH*-*CFHR1*, causing atypical hemolytic uremic syndrome has also been reported before [25]. This deletion, with breakpoints within the last three exons of *CFH* and *CFHR1*, results in a hybrid *CFH*-*CFHR1* protein, which leads to atypical hemolytic uremic syndrome as a result of defective recognition function.

Our analysis of the reference HapMap samples and several large clinical datasets suggests that the critical AMD region of risk spans 32 kb that is not dependent on the presence of rs1061170. Based on haplotypes derived from five SNPs, Li et al. [16] showed that ∼6% of risk haplotypes lack the “C” allele at codon 402. We conducted haplotype analysis using 78 informative SNPs and found the H6 haplotype, which bore a recombinant that separated codon 402 from the core risk haplotype. The plausibility of rs1061170 as the AMD causal variant was originally put forward by Edwards et al. [4] and Haines et al. [8] when high resolution arrays with copy number information were not commercially available. This variant remained a strong contender because of its efficacy as a tag, and the weight of the biological evidence, e.g., due to its interaction with C-reactive protein, an important specific marker of inflammation [28]. Recently, Hakobyan et al. [29] developed monoclonal antibodies for allele-specific factor H levels assayed in plasma. Their data show that factor H levels increase with age, but that there is lack of correlation between plasma levels by genotype and AMD status; the authors attribute these discrepancies to other factors e.g. smoking. We also observed no evolutionary conservation either at tyrosine or histidine when comparing the orthologous sequence (Figure S9). In considering the sum of evidence, we put forward that rs1061170 may only be causal in the context of the 32 kb critical region, which harbors the two best signals at rs1329428 and rs203687.

An interesting feature of the 32 kb sequence is that it can be partitioned into a 12 kb segmentally duplicated block and a 20 kb unique region. rs203687 maps to the segmentally duplicated block, while rs1329428 maps to the unique sequence. The segmental duplication shows 89% homology with the noncoding region between *CFHR1* and *CFHR4* which contains ΔCNP 148, suggesting that the protection offered by this deletion variant may be directly mediated through this similarity. The LD between rs1329428 and rs203687 is modest (r^2^ = 0.57). rs2274700, earlier reported by Li et al. [16], localizes to the segmental duplication block, while rs1410996, reported by Maller et al. [17], maps to the unique fragment at intron 14. In the HapMap CEU data, the LD between rs1329428 and rs1410996 is perfect (r^2^ = 1). The results show an equally strong signal at rs1410996, and is not feasible to statistically determine which variant is causal. The region with the best signal contains complement control protein modules 12–14, a putative C3b/C3c binding site [30]. The important finding is that the core sequence containing the AMD susceptibility variant(s) has a complex structure affecting both copy number and gene regulation.

In summary, we have expanded the knowledge base of the spectrum of genetic variation at the *CFH* locus and its paralogs; *CFH* was the first gene with a common variant to be identified through GWAS. As with loci that function in immunity cascades, these genes show signs of positive selection and recurrent rearrangements, an attribute of CNVs. We also circumscribed a critical region within *CFH* that carry AMD risk-causing variants. The AMD field has begun to apply the original gene-mediated risk profiles towards personalized medicine. Maller et al. [17] proposed assessing lifetime risk of AMD by evaluating five common SNPs at *CFH*, *ARMS2*, *C2* and *CFB*, and ranking risk by counting the number of susceptibility variants at each locus. Seddon et al. [31] have shown that the predictive power for AMD progression at six SNPs in these genes along with demographic profiles is over 82%. However, our results show that common and rare deletions, duplications, and rearrangements are complex phenomena that may lead to unanticipated results, particularly when the net consequence of the diplotype across both homologs is taken into consideration. *CFH* has similarly been the focus of examination for atypical hemolytic uremic syndrome and Membranoproliferative Glomerulonephritis type II (MPGN II). Expansion of the repertoire of variation at the expanded RCA locus will have an impact on the predictive ability for both diseases, AMD and MPGN/atypical hemolytic uremic syndrome, with more comprehensive models being required for forecasting future disease status. We suggest that a systematic survey of larger clinical datasets is needed to understand the role of these common and rare events in mediating susceptibility and protection.

## Materials and Methods

### Ethics Statement

This study was approved by the Institutional Review Boards of the Case Western Reserve University, Cleveland; University of Wisconsin, Madison; University of Michigan, Ann Arbor; Cleveland VA Medical Center, Cleveland; Cleveland Clinic Foundation; Cleveland; Mayo Clinic, Rochester; University of Pennsylvania, Philadelphia; Oregon Health & Sciences University, Portland; Singapore Eye Research Institute, Singapore; and ethics sub-committee of the Vision Research Foundation, Chennai, India.

### Subjects

#### Reference Sets

The reference sets include 270 phase I+II HapMap samples (30 Utah residents of Northern and Western European ancestry (CEU) trios, 30 Yoruba in Ibadan (YRI) trios, 45 Han Chinese in Beijing (CHB) and 45 Japanese in Tokyo (JPT)) and 912 HapMap phase III samples, belonging to 11 different populations (CEU, CHB, JPT, YRI, Chinese in Denver, Gujarati Indians in Houston, Luhya in Webuye, Kenya, Mexicans in Los Angeles, Maasai in Kinyawa, Kenya, Tuscans in Italy and Africans in Southwest USA. We also examined 400 samples, comprising 100 each of African Americans, Caucasians, Chinese and Mexicans, belonging to the Human Variation Panel from the Coriell Institute for Medical Research (Coriell, NJ, USA). The raw intensity files (Affymetrix genome-wide Human SNP6.0 array) of all these samples were obtained directly from Affymetrix (Santa Clara, CA), http://www.hapmap.org and the Coriell Institute for Medical Research (Camden, NJ), respectively.

#### Clinical Sets

Our clinical datasets include 293 individuals from 34 families participating in Familial Age-related Maculopathy Study (FARMS), 600 cases and controls (400 cases and 200 controls) participating in the Age-related Eye disease study (AREDS), a Michigan-Penn-Mayo (MPM) Panel containing of 2157 cases and 1150 controls, a South Indian cohort with 251 individuals (134 cases and 117 controls) and the Singapore Malay Eye Study (SiMES) cohort containing 3072 samples on whom GWAS data was available (Figure S10 and Table S8 for details of cohort phenotyping and genotyping). In addition to the GWAS data, a panel of 1941 severe cases and 1991 controls from five different cohorts (referred to as the custom Illumina panel) were also examined using custom genotyping. The description of the clinical sets is presented in Text S2 and Table S8. This study was conducted according to the principles expressed in the Declaration of Helsinki. All samples were collected according to institutionally approved protocols for study of human subjects at the respective Institute and written informed consent was obtained from all subjects.

### Sequence Similarity Search

To detect similar segments in the RCA gene cluster, we obtained genomic sequence of this region (NCBI build 36; http://www.ensembl.org) and used Megablast search option in the Basic Local Alignment Search Tool (BLAST) at NCBI. In primates, the segmental duplications were detected using the whole-genome shotgun sequence detection approach [32]. The strategy entails the alignment of orangutan and chimpanzee whole genome shotgun reads using the Megablast program and identifying regions with a statistically significant excess read depth (see Marques-Bonet et al. [33] for details).

### Genotyping and Quality Control

The DNA samples used in this study were genotyped with a variety of platforms. The genotyping methods as well as quality control criteria are described in Table S8.

#### CFH rs1061170 genotyping

This variant of *CFH* was not placed in the whole genome arrays or in the GoldenGate custom Illumina genotyping array. Thus, this variant was genotyped in the FARMS, AREDS, the custom Illumina panel and Indian cohort using a custom assay (Applied Biosystems, Foster City, CA) or direct sequencing. Results for the FARMS rs1061170 data are published elsewhere [10], [34].

### Copy Number Variation (CNV) Detection

Copy number variant detection was performed by different methods depending on the available genotyping platform (Table S8). The common and rare copy number variations were detected using the Birdsuite software version 1.5 [35], [36] (Broad Institute, Boston, MA) in both the reference and the FARMS clinical sets. The raw intensity data from the AREDS as well as the Indian cohort were analyzed using Partek Genomic Suite Version 6.4 (Partek Inc., MO, USA). The PennCNV algorithm [37] was used to detect CNVs on the Illumina Bead Array data, e. g., the MPM panel, the SiMES cohort and the custom Illumina panel.

Fosmid clones corresponding to CNP147 and CNP148 were identified from the fosmid Human Genome Structural Variation Project [38] based on the mapping of end-sequence pairs to the regions (http://hgsv.washington.edu). Sites of structural variation were confirmed by array comparative genomic hybridization using corresponding test HapMap samples and the reference genome of sample NA15510 [36]. Clones were recovered and inserts were completely sequenced using Sanger-based capillary sequencing [39]. The region between *CFH* and *ASPM* was screened using polymerase chain reaction (PCR) to molecularly validate the homozygous deletions detected by the Birdsuite program, and to find additional large homozygous deletions, if any (Figure S11). Primers were designed either manually or using Primer3 software (http://frodo.wi.mit.edu/primer3/). We amplified a total of 30 fragments between *CFH* and *ASPM* either as single fragments or multiple fragments using standard PCR with DNA from 10 HapMap CEU samples. We also screened 98 samples (49 Caucasians and 49 African Americans) from the Human Variation panel, the entire FARMS cohort, and 443 of 511 AREDS samples that were genotyped with both the Affymetrix 100 K and the Illumina 100 K arrays.

### Statistical Analysis

#### Haplotype Construction

We downloaded phase II HapMap data for 305 SNPs located between CFH and CFHR5. A single JPT sample (NA19012) with a large number of missing genotypes in this region was not considered for haplotyping due to poor data structure. Haplotypes, inferred using PHASE 2.2.1 [40], [41] with a population frequency of >0.5% were considered further. Identical haplotypes were assigned the same number and all the haplotypes were categorized in the order of similarity with an unweighted group pairs method that uses arithmetic averages (UGPMA) using the Molecular Evolutionary Genetics Analysis software version 4.0 (MEGA4) [42].

For the FARMS cohort, the haplotypes were constructed manually according to the segregation pattern and missing alleles were imputed by a parsimony method. After this, Mendelian errors were identified at the haplotype level using MARKERINFO (S.A.G.E. version 5.3) and haplotypes with a frequency of >1% were considered for further analysis. For the case-control cohorts, two most likely PHASE 2.1.1 inferred haplotypes were selected from each individual and haplotypes with a frequency of >1% were examined further.

#### Linkage Disequilibrium (LD) Analysis in HapMap Samples

We examined the LD structure between these CNPs and the SNPs located at the RCA gene cluster using Haploview version 4.0 [43]. For this, we picked the two most likely haplotypes inferred by PHASE from each of 209 unrelated HapMap samples.

#### Imputation

To maximize the number of available markers, we imputed the untyped genotypes using the MACH program version 1.0 [44] with HapMap CEU haplotypes as the reference to obtain much denser data.

#### Association Analysis

The SNPs located between *CFH* and *ZBTB41* that passed quality control metrics were tested for association, with the minor allele as reference. For the deletion variants and the haplotypes, the total number of copies present (N = 0, 1 or 2) was tested against the affection status. When rs1329428 and rs203687 were included in association models as SNP covariates, they were coded in a similar manner. For the FARMS cohort, the original 15-level phenotype measurement of Wisconsin age-related maculopathy grading system (WARMGS) score was adjusted to age 80 using a Box-Cox transformation method as previously described [2]. To account for the linear and non-linear effects of age, we adjusted for both age and age^2^ (age squared). Family-based association analysis on the FARMS data was conducted using the age-adjusted scores as the disease trait using the ASSOC program (S.A.G.E. version 5.3), with each SNP was tested for association under the additive mode of inheritance in the presence of a random sibship effect. Single SNP association analysis for the MPM panel is described elsewhere [45]. For other case-control cohorts, logistic regression, as implemented in PLINK [46], was used to test the association of SNPs (covariates used are described in Table S8). For haplotype association analysis, the number of copies of every haplotype except H2 were included simultaneously using H2 as baseline and this results in estimates of the effect of each haplotype relative to the base haplotype H2.

Since cases and controls included in the custom Illumina panel were identified at different sites over differing time frames, we were concerned about heterogeneity. Therefore, each cohort was analyzed separately and the results combined using meta-analysis. This approach is more conservative than a pooled analysis grouping all cases and controls. To combine findings from different cohorts, a joint effect estimate was calculated by taking a weighted average of the individual cohort estimates, with the inverse of the variance as the weighting factor. This combined estimate was used to determine an overall odds ratio for each SNP and its standard error, from which the p-value was calculated assuming normality of the distribution.

#### Finding an Optimal SNP Set

In order to rank the best SNPs in the 32 kb region and to conduct a minimal SNP set explaining the effect of the region on AMD, we ran forward selection. SNP genotypes (imputed) in the pooled set containing the three Caucasian case-control datasets (the custom Illumina panel, the MPM panel and the AREDS cohort) were coded as the number of minor alleles, and were added one at a time as predictors in a logistic regression model, including age and sex as covariates. At each step, the SNP giving the smallest p value was retained in the model, until no additional SNP remained significantly associated (p<0.05).

### Functional Analysis

We used two programs, AliBaba version 2.1 (http://www.gene-regulation.com/pub/programs.html) and Eldorado (Genomatix software Inc. MI, USA) to examine the two important intronic SNPs in the 32 kb core region. These programs enable us to predict if changes in the nucleotide at this position, alters the binding properties of the transcription factors. The amino acid sequence around codon 402 of human *CFH* protein was compared to that of other species using protein blast at the NCBI website.

### Gene Expression Analysis

We downloaded gene expression data for Epstein-Barr Virus transformed B cell lines (GENEVAR project, http://www.sanger.ac.uk/humgen/genevar/) of 210 unrelated HapMap individuals, which consisted of 60 parents of CEU, 45 CHB, 45 JPT, and 60 parents of YRI. We examined the effect of AMD-associated SNPs on expression of *CFH* and *CFH*-related genes, *CFHR1-CFHR5*. To avoid population stratification due to differences in allele frequencies among different ethnic groups, we tested association separately in each population using linear regression. Effects (regression coefficients) and p- values from regression results were combined by meta-analysis using the METAL program (http://www.sph.umich.edu/csg/abecasis/Metal/index.html), which calculates an overall Z-statistic and p-value from the weighted average of the individual statistics by accounting for sample size in each sample.

## Supporting Information

Figure S1 Segmental duplications in the RCA gene cluster. (**A**) Predicted/known segmental duplications as shown in UCSC human genome browser (NCBI Build 36, as of December 2008) as well as in the literature. Homologous regions are indicated by horizontal shaded bars of the same color. (**B**) Predicted segmental duplications based on sequence similarity as shown by megablast (NCBI). Highly similar regions are indicated with colored bars (see **Table S1** for more detail). The homologous regions encompassing sequences identical to Y402H of *CFH* and the surrounding region are shown in red bars. A major proportion of SNPs (2043/2771) located at RCA gene clusters fall within these segmental duplications.
(TIF)

Figure S2 Depth of coverage analysis of whole genome shotgun sequence aligned to the reference genome predicts Venter may have partial deletion of *CFHR1* when compared to Watson, but Watson has a predicted duplication of *CFHR4* (regions in red depict areas of excess depth of coverage).
(TIF)

Figure S3 Confirmation of homozygous deletions at CNP147 using the PCR-based deletion screening protocol (see **Figure S11** for primers location). Gel picture of fragment Unique 01 showing no band in HapMap CEU samples (**a**) and in samples from Coriell human diversity panel (**b**) predicted to have both copies of CNP147 deleted.
(TIF)

Figure S4 Arrangement of haplotypes derived from three different HapMap groups in the order of similarity. Haplotypes highlighted with yellow are the ones carrying deletion either at CNP147 or CNP148. All the haplotypes are categorized according to haplogroups observed in our clinical datasets (see figure 2 in the main paper for more information). ΔCNP147 occurs on multiple clades in the Yorubans, suggesting recurrent origin. Although this deletion occurs on two different clades in CEU, they appeared to be separated by recombination event within the CNP148 region. ΔCNP148 occurs on multiple haplotype background both in Asians and Yorubans.
(DOC)

Figure S5 PCR amplification of fragments (**a**) Unique 01, (**b**) Frag_00.7.4 & Frag_B1_2.2, and (**c**) Frag_R3.05 & Frag_R1.13 confirming homozygous deletions in FARMS samples.
(TIF)

Figure S6 Gel pictures of fragments. ΔCNP147 carrying haplotypes were predicted using allele “A” of rs2019727 because none of the deletion-specific SNPs were genotyped in AREDS. rs2019727 is in very strong LD with ΔCNP147 (r^2^ = 0.9) in the Caucasian reference sets. Predicted homozygous deletions at CNP147 in AREDS samples were confirmed using PCR-based deletion screening protocol. Amplification of fragments (**a**) Unique 01 and (**b**) Frag_R3.05 & Frag_R1.13 confirmed the homozygous deletions in AREDS samples (as indicated by arrows).
(TIF)

Figure S7 Locations of the *Cis*-elements, as predicted by chromatin immunoprecipitation assay, at *CFH* locus. This data was obtained from UCSC Human genome browser. Arrow indicates the transcriptional binding sites detected at rs203687 and rs1329428.
(TIF)

Figure S8 Comparative primate genome analysis (depth of coverage and interspecific arrayCGH) suggests that chimps have more extensive duplication architecture than humans, and that duplications likely arose in a common ancestor of chimp and human but after divergence from orangutan (6–12 million years ago). Targeted arrayCGH of the region confirms increase of *CFHR* copies in chimpanzee (PTR) and bonobo (PPA) when compared to human but that orangutan (PPY) has reduced copy-number. Interestingly, a portion of *CFHR4* seems to have hyperexpanded in great apes (bonobo and orangutan).
(TIF)

Figure S9 Evolutionary conservation of codon 402 of *CFH*. Neither Tyrosine nor Histidine at codon 402 is conversed in other species. Arrow shows the codon 402 of *CFH*.
(TIF)

Figure S10 Study design. This study consisted of several stages. In the first stage, structural variation at RCA gene cluster were detected in the reference sets. The variations found in >1% of the population, called copy number polymorphisms (CNP), were characterized. In the second stage the significance of these CNPs on AMD was examined in the clinical sets. In the third stage, haplotypes were constructed using CNP and SNP genotypes and the haplotypes over 1% frequency were tested for association with AMD. Information from these haplotypes was used to fine map the critical region. In the fifth stage, best SNP was selected from this critical region by conditional analysis. In addition, we also tested effect of these CNPs and AMD-associated SNPs on the expression of genes involved in complement regulation. Finally, functional studies were conducted for one of the AMD-associated SNP affecting the *CFH* gene expression.
(TIF)

Figure S11 Schematic representation of PCR-based deletion screening plan. The region of interest was divided into three subregions, 1–3. Subregion 1 spanned CNP147; subregion 2 spanned the region between the 5′ end of the *CFH* gene and 5′ end of CNP147; and subregion 3 was defined as the region from the 3′ end of CNP147 to the middle of *ASPM*. (**a**) Enlargement of region around unique region 01. Megablast search at CNP147 revealed a unique region that did not show homology to any other regions in the genome. It is located at 195,066,977–195,010,019. (**b**) Arrangement of genes in the RCA gene cluster, with the arrow indicating the 5′-3′ direction. The vertical arrows indicate the locations screened; three subregions are indicated with different colors of arrows: blue arrows represent the subregion 01, which includes CNP147. Subregions 2 and 3 are indicated with black and red arrows, respectively.
(TIF)

Table S1 Location of highly similar segments in the RCA gene cluster, as identified through Megablast search.
(XLS)

Table S2 Rare structural variations detected among reference sets.
(XLS)

Table S3 Frequency distribution of variations at CNP147 and CNP148 in different reference populations.
(XLS)

Table S4 Association of six different haplotypes of CFH region with AMD in the custom illumina panel.
(XLS)

Table S5 Meta-analysis SNPs located in the 32 kb critical region.
(XLS)

Table S6 Variations at CNPs 147 and 148 detected with PennCNV in the custom illumina panel.
(XLS)

Table S7 Rare structural variations in the RCA gene cluster in FARMS cohort.
(XLS)

Table S8 Summary of SNP genotyping, genotype calling and quality control measures used in this study.
(XLS)

Text S1 BLAST alignment of sequences surrounding some of the AMD-associated SNPs in the RCA gene cluster with their paralog sequences.
(DOC)

Text S2 Supplementary methods.
(DOC)
