# Correction: A 32 kb Critical Region Excluding Y402H in *CFH* Mediates Risk for Age-Related Macular Degeneration

**DOI:** 10.1371/journal.pone.0209943

**Published:** 2018-12-20

**Authors:** Theru A. Sivakumaran, Robert P. Igo, Jeffrey M. Kidd, Andy Itsara, Laura J. Kopplin, Wei Chen, Stephanie A. Hagstrom, Neal S. Peachey, Peter J. Francis, Michael L. Klein, Emily Y. Chew, Vedam L. Ramprasad, Wan-Ting Tay, Paul Mitchell, Mark Seielstad, Dwight E. Stambolian, Albert O. Edwards, Kristine E. Lee, Dmitry V. Leontiev, Gyungah Jun, Yang Wang, Liping Tian, Feiyou Qiu, Alice K. Henning, Thomas LaFramboise, Parveen Sen, Manoharan Aarthi, Ronnie George, Rajiv Raman, Manmath Kumar Das, Lingam Vijaya, Govindasamy Kumaramanickavel, Tien Y. Wong, Anand Swaroop, Goncalo R. Abecasis, Ronald Klein, Barbara E. K. Klein, Deborah A. Nickerson, Evan E. Eichler, Sudha K. Iyengar

The authors have stated that the coordinates of two deletions of the CFHR gene cluster on chromosome 1 represent the outer boundaries of the deletion interval based on patterns of sequence identity and included both copies of the duplicated sequence where breakpoint definition is difficult. For CNP147, this interval of 86.302kbp (chr1: 194988828–195075129) includes 1617 bp of 100% identical sequence located at the 5’ and 3’ breakpoints. Since the deletion allele retains a single copy of this sequence by virtue of non-allelic homologous recombination, the CNP147 deletion event in fact removes 84.685 kbp, a size consistent with the findings of Hughes et al. (Hughes et al Nature Genetics, volume 38, 1173–1177, 2006). Similarly, the reported boundaries for CNP148 with an interval of 121.959 kbp (chr1: 195049336–195171294) include a 59 bp segment of identical sequence at each breakpoint and therefore results in a loss of 121.9 kbp. The CNP148 breakpoints are further embedded within an extended segment of high sequence identity (> 1kbp and 99% identity). The authors would like to make multiple corrections.

There is an error in the ninth sentence of the “Structural Variation at the RCA Gene Cluster” section within the Results. The correct sentence is: The deletion at CNP147 was 84.685 kb (chr1: 194,988,828–195,073,512) consistent with previous reports (Hughes et al. 2006).

There are multiple errors in the caption for Fig 2. Please see the corrected Fig 2 caption here.

**Fig 2:**
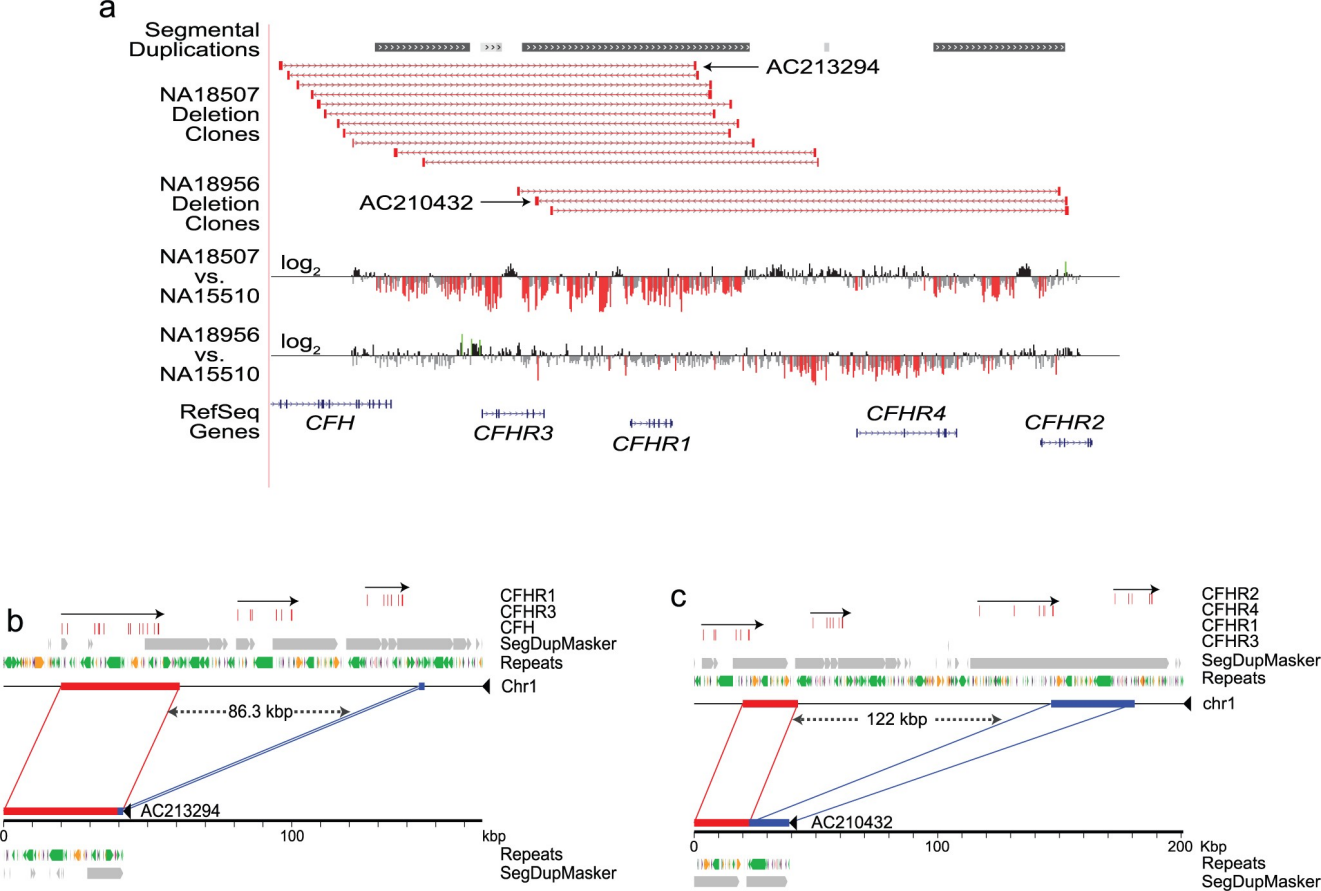
Validation of CNPs in the HapMap samples. (**2a**) UCSC browser view (http://humanparalogy.gs.washington.edu/) showing organization and structural variation in the RCA gene cluster. Red bars indicate the sites of structural variation in eight individuals underwent fosmid pair-end sequencing. Deletions at CNP147 and CNP148 were using high-resolution tiling-path custom array-based CGH. Probes with log2 ratios below or above a threshold of 1.5 s.d. from the normalized mean log2 ratio are colored red (deletions) or green (duplication), respectively. (**2b**) and (**2c**) Alignment of sequenced fosmid inserts against the human genome assembly (build36) confirms the extent of two deletions in the CFHR cluster on chromosome 1. Clone AC213924, derived from sample NA18507, corresponds to deletion CNP147 (**2b**). This variant removes 84.685 kbp of sequence from an 86.302 kbp interval (chr1:194,988,828–195,075,129, build36), while retaining one copy of a 1,617 bp segment of identical sequence found at each breakpoint and resulting in loss of the *CFHR3* and *CFHR1* genes. Clone AC210432, derived from sample NA18956, corresponds to deletion CNP148 (**2c**). This variant removes 121.9 kbp of sequence from a 121.959 kbp interval (chr1:195,049,336–195,171,294), while retaining one copy of a 59 bp segment found at both breakpoints. The CNP148 breakpoints are further embedded within an extended stretch of high sequence identity (>1kbp; >99%). Please note that in (**2b**) and (**2c**) the lengths of the deletion intervals for CNP147 and CNP148 are rounded to one decimal place.
