# Supplementary figures and images for: A 32 kb Critical Region Excluding Y402H in CFH Mediates Risk for Age-Related Macular Degeneration
Source: PLoS One. 2011 Oct 12;6(10):e25598. doi: 10.1371/journal.pone.0025598 (PMC3192039; doi:10.1371/journal.pone.0025598)

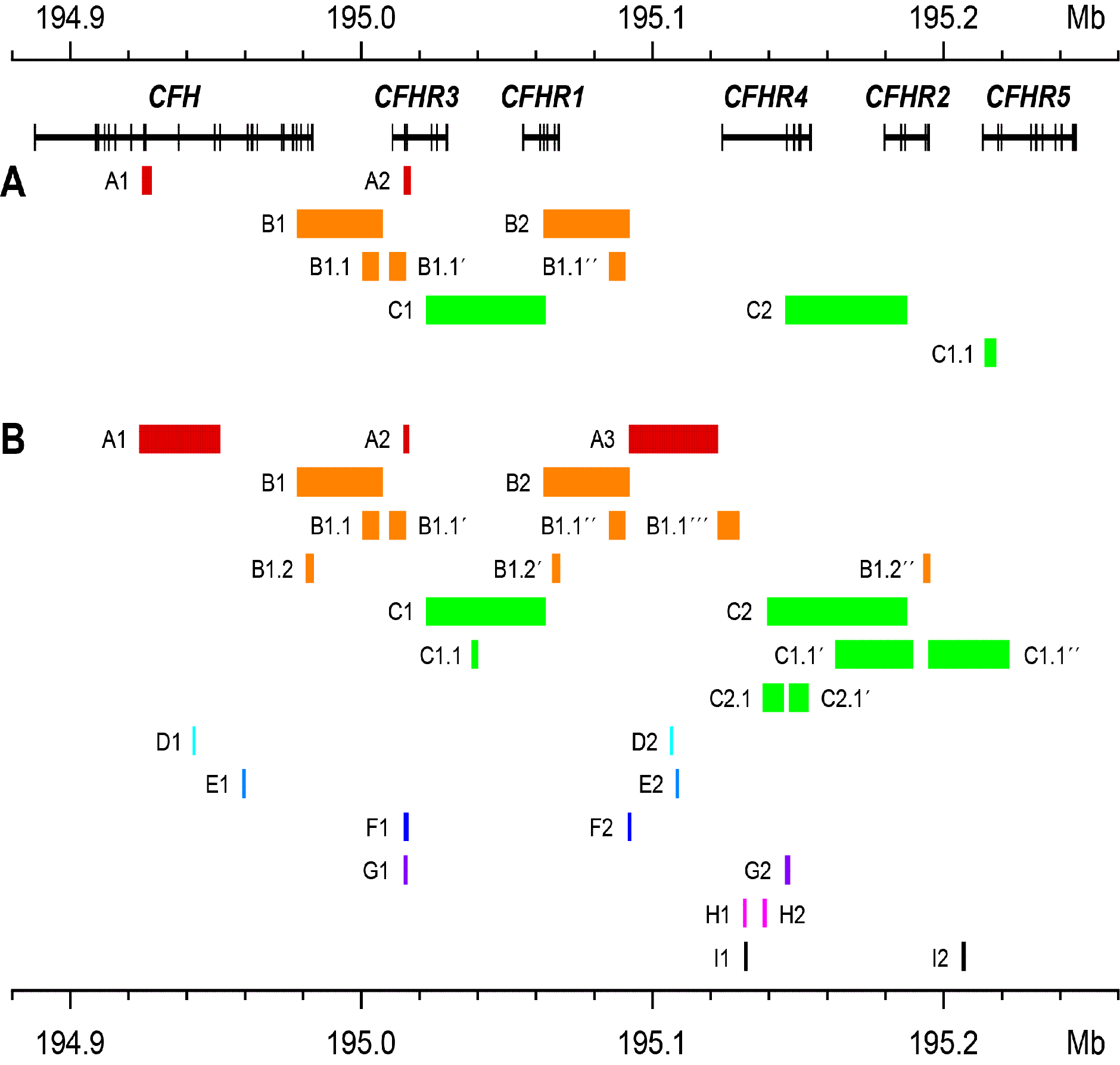

Supplement: Figure S1 — Segmental duplications in the RCA gene cluster. (A) Predicted/known segmental duplications as shown in UCSC human genome browser (NCBI Build 36, as of December 2008) as well as in the literature. Homologous regions are indicated by horizontal shaded bars of the same color. (B) Predicted segmental duplications based on sequence similarity as shown by megablast (NCBI). Highly similar regions are indicated with colored bars (see Table S1 for more detail). The homologous regions encompassing sequences identical to Y402H of CFH and the surrounding region are shown in red bars. A major proportion of SNPs (2043/2771) located at RCA gene clusters fall within these segmental duplications. (TIF) [file pone.0025598.s001.tif]

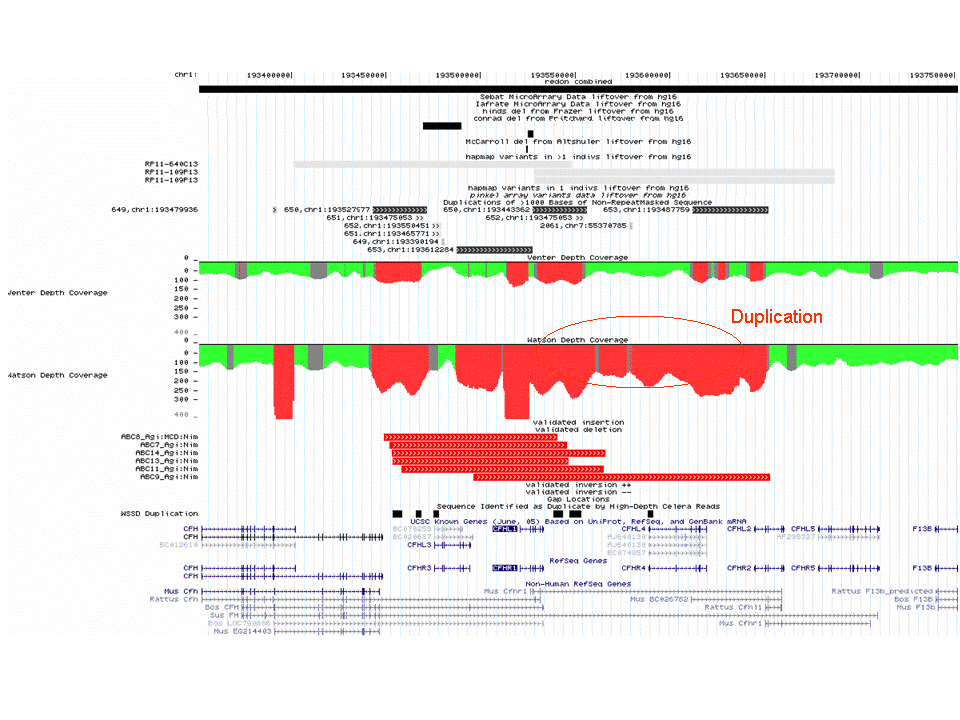

Supplement: Figure S2 — Depth of coverage analysis of whole genome shotgun sequence aligned to the reference genome predicts Venter may have partial deletion of CFHR1 when compared to Watson, but Watson has a predicted duplication of CFHR4 (regions in red depict areas of excess depth of coverage). (TIF) [file pone.0025598.s002.tif]

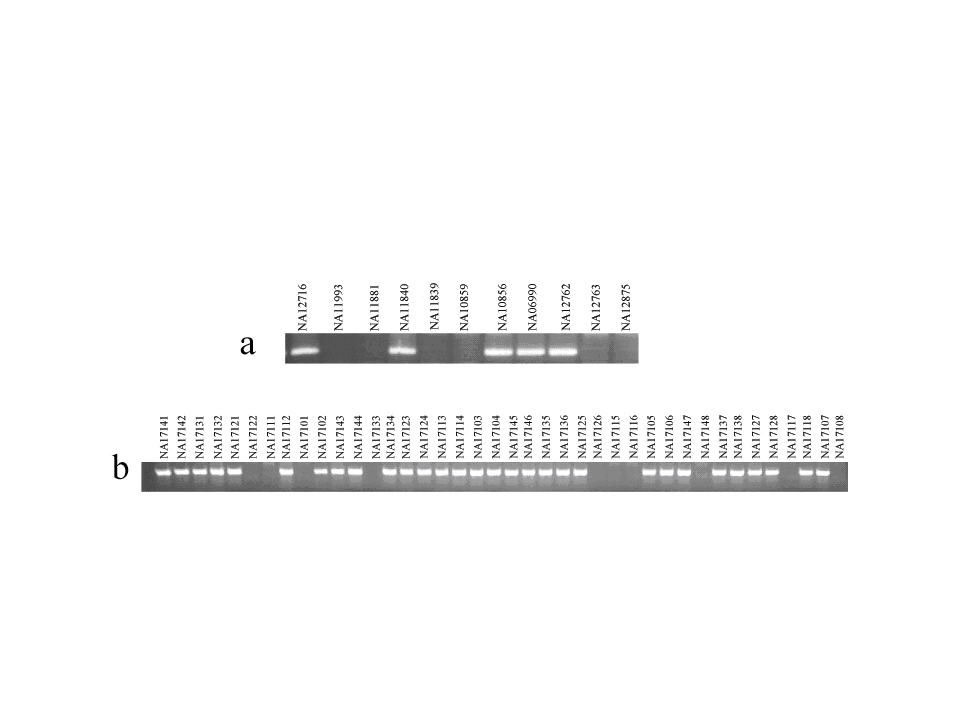

Supplement: Figure S3 — Confirmation of homozygous deletions at CNP147 using the PCR-based deletion screening protocol (see Figure S11 for primers location). Gel picture of fragment Unique 01 showing no band in HapMap CEU samples (a) and in samples from Coriell human diversity panel (b) predicted to have both copies of CNP147 deleted. (TIF) [file pone.0025598.s003.tif]

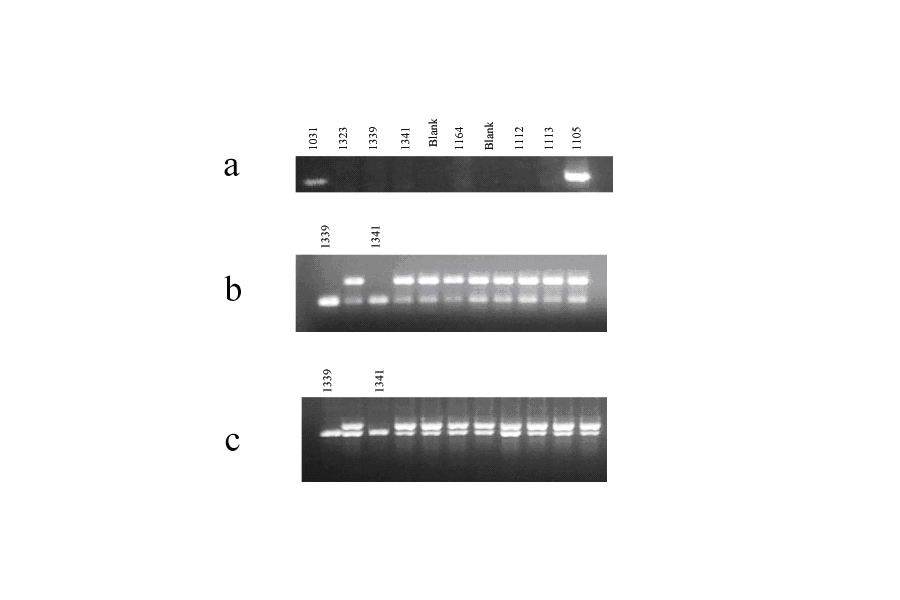

Supplement: Figure S5 — PCR amplification of fragments (a) Unique 01, (b) Frag_00.7.4 & Frag_B1_2.2, and (c) Frag_R3.05 & Frag_R1.13 confirming homozygous deletions in FARMS samples. (TIF) [file pone.0025598.s005.tif]

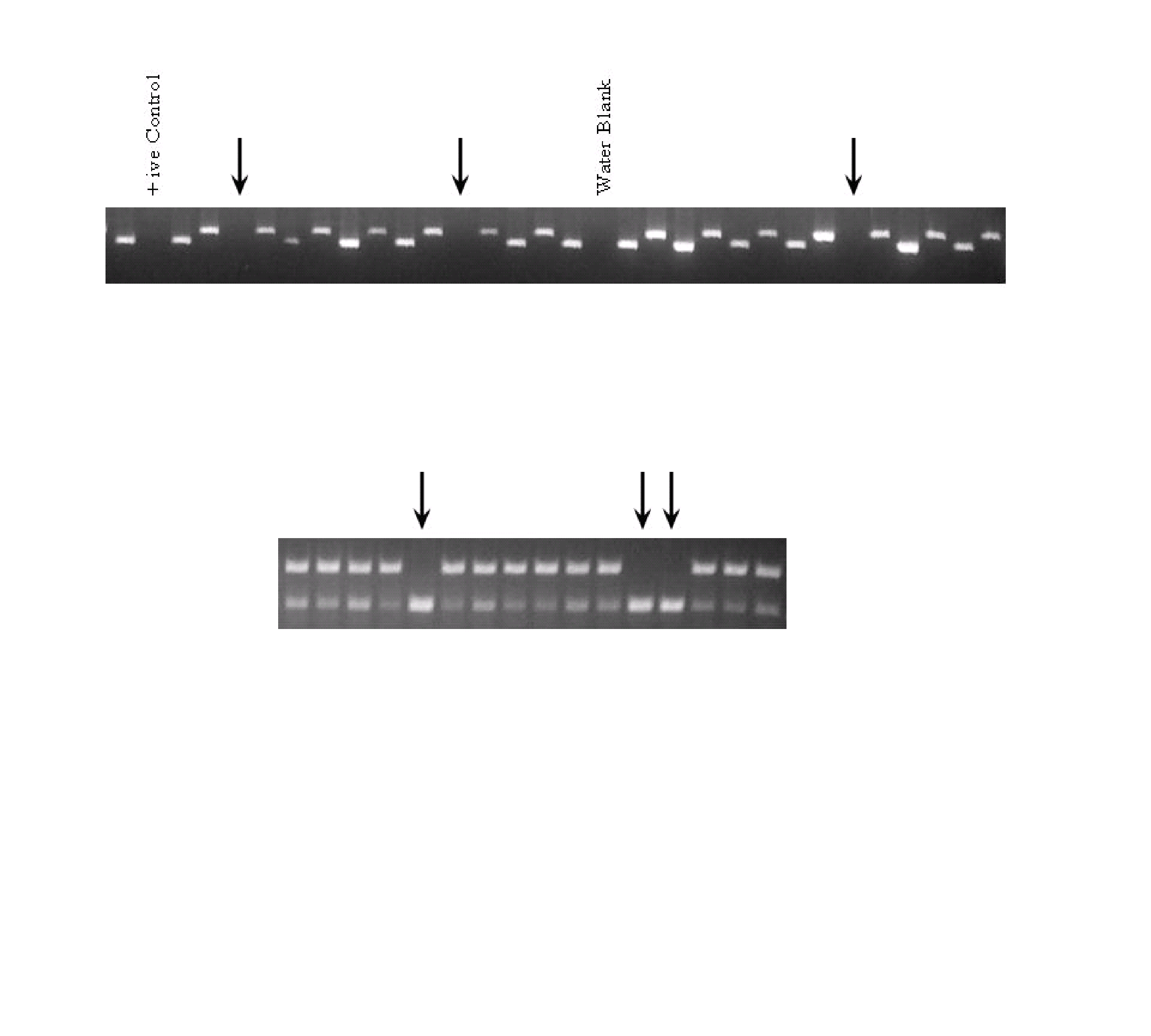

Supplement: Figure S6 — Gel pictures of fragments. ΔCNP147 carrying haplotypes were predicted using allele “A” of rs2019727 because none of the deletion-specific SNPs were genotyped in AREDS. rs2019727 is in very strong LD with ΔCNP147 (r2 = 0.9) in the Caucasian reference sets. Predicted homozygous deletions at CNP147 in AREDS samples were confirmed using PCR-based deletion screening protocol. Amplification of fragments (a) Unique 01 and (b) Frag_R3.05 & Frag_R1.13 confirmed the homozygous deletions in AREDS samples (as indicated by arrows). (TIF) [file pone.0025598.s006.tif]

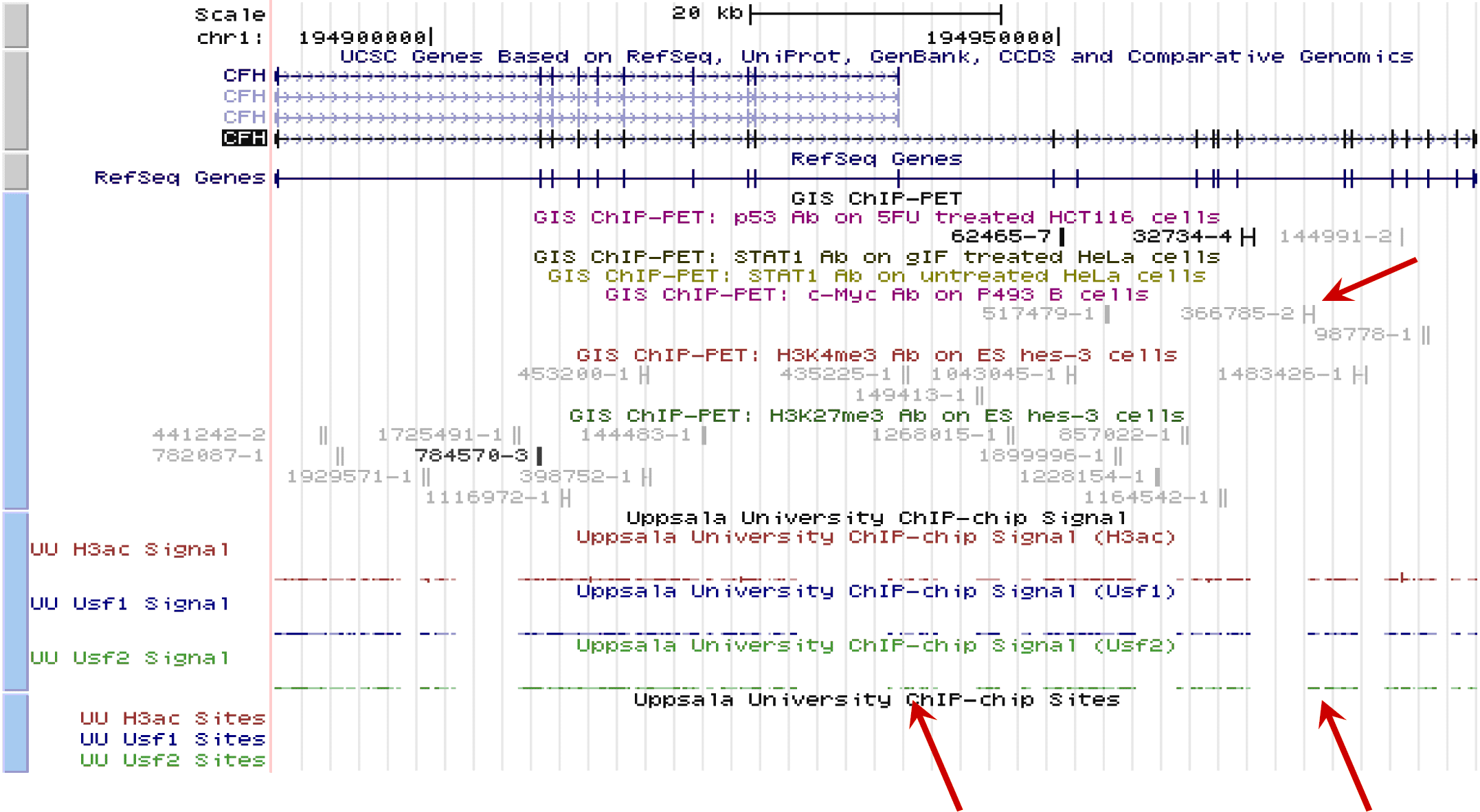

Supplement: Figure S7 — Locations of the Cis-elements, as predicted by chromatin immunoprecipitation assay, at CFH locus. This data was obtained from UCSC Human genome browser. Arrow indicates the transcriptional binding sites detected at rs203687 and rs1329428. (TIF) [file pone.0025598.s007.tif]

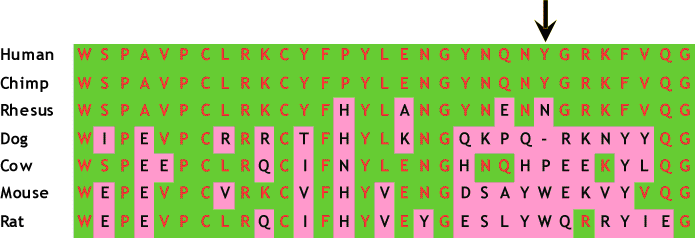

Supplement: Figure S9 — Evolutionary conservation of codon 402 of CFH. Neither Tyrosine nor Histidine at codon 402 is conversed in other species. Arrow shows the codon 402 of CFH. (TIF) [file pone.0025598.s009.tif]

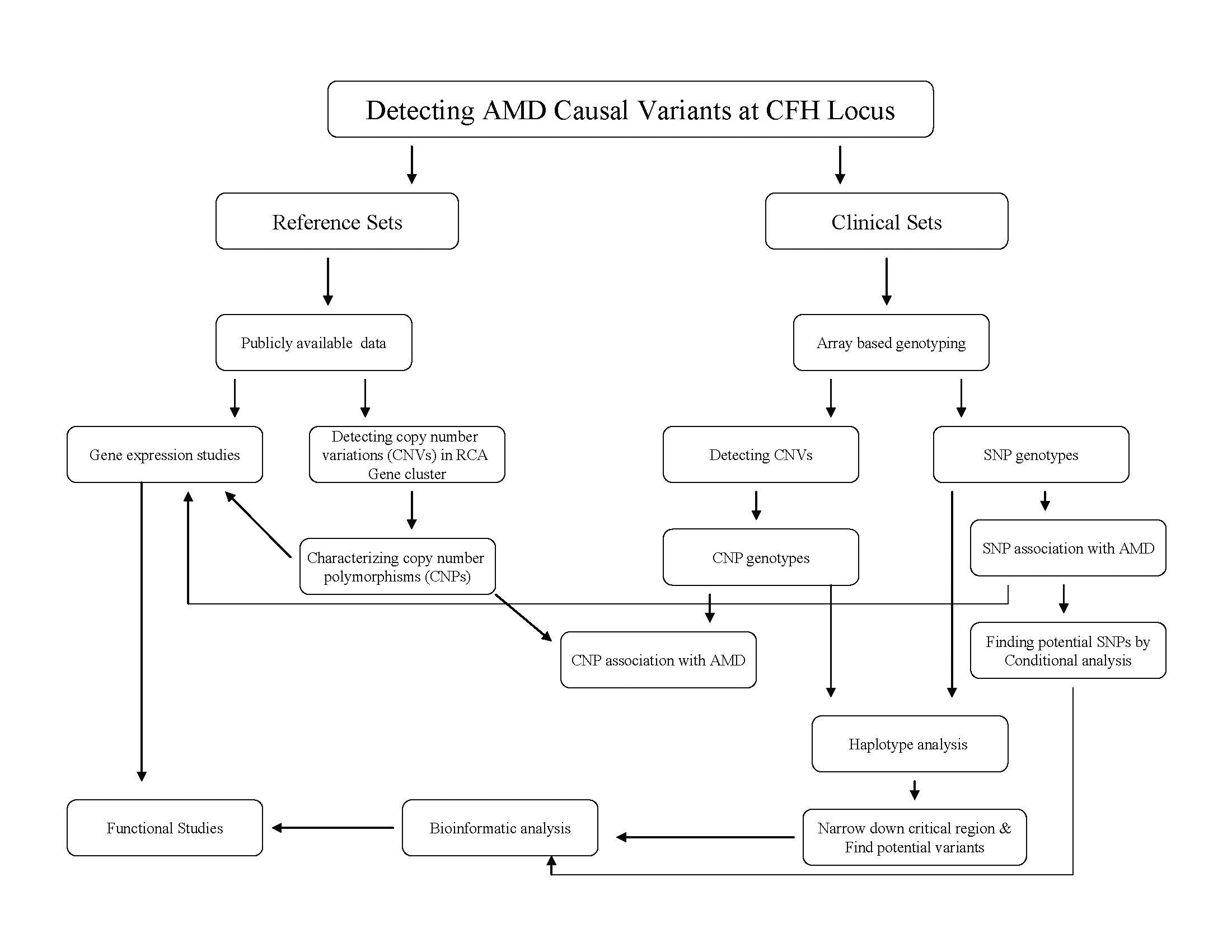

Supplement: Figure S10 — Study design. This study consisted of several stages. In the first stage, structural variation at RCA gene cluster were detected in the reference sets. The variations found in >1% of the population, called copy number polymorphisms (CNP), were characterized. In the second stage the significance of these CNPs on AMD was examined in the clinical sets. In the third stage, haplotypes were constructed using CNP and SNP genotypes and the haplotypes over 1% frequency were tested for association with AMD. Information from these haplotypes was used to fine map the critical region. In the fifth stage, best SNP was selected from this critical region by conditional analysis. In addition, we also tested effect of these CNPs and AMD-associated SNPs on the expression of genes involved in complement regulation. Finally, functional studies were conducted for one of the AMD-associated SNP affecting the CFH gene expression. (TIF) [file pone.0025598.s010.tif]

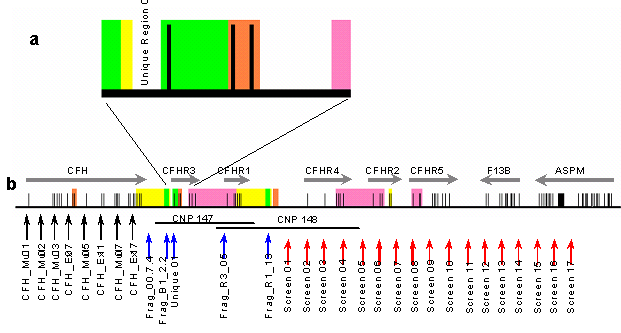

Supplement: Figure S11 — Schematic representation of PCR-based deletion screening plan. The region of interest was divided into three subregions, 1–3. Subregion 1 spanned CNP147; subregion 2 spanned the region between the 5′ end of the CFH gene and 5′ end of CNP147; and subregion 3 was defined as the region from the 3′ end of CNP147 to the middle of ASPM. (a) Enlargement of region around unique region 01. Megablast search at CNP147 revealed a unique region that did not show homology to any other regions in the genome. It is located at 195,066,977–195,010,019. (b) Arrangement of genes in the RCA gene cluster, with the arrow indicating the 5′-3′ direction. The vertical arrows indicate the locations screened; three subregions are indicated with different colors of arrows: blue arrows represent the subregion 01, which includes CNP147. Subregions 2 and 3 are indicated with black and red arrows, respectively. (TIF) [file pone.0025598.s011.tif]
