# Supplementary material for: A 32 kb Critical Region Excluding Y402H in CFH Mediates Risk for Age-Related Macular Degeneration
Source: PLoS One. 2011 Oct 12;6(10):e25598. doi: 10.1371/journal.pone.0025598 (PMC3192039; doi:10.1371/journal.pone.0025598)
Supplement: Text S1 — BLAST alignment of sequences surrounding some of the AMD-associated SNPs in the RCA gene cluster with their paralog sequences. (DOC) [file pone.0025598.s020.doc]

**Text S1. BLAST alignment of sequences surrounding some of the AMD-associated SNPs in the RCA gene cluster with their paralog sequences.**

**rs7535263 (Position: 194,948,969; Intron 9)**

**Query 194,948,842 - 194,949,079; Subject 195,119,712 - 195119949**

Query 61842 AACCGACAGTAATTTCTATCATCAGTTAACAGAAACAGTAACT-TCCACTCTTTCAAGTT 61900

|||| |||||||||||||||||||||| ||||| ||||||| | ||||||||||||||||

Sbjct 47343443 AACCAACAGTAATTTCTATCATCAGTTTACAGAGACAGTAA-TGTCCACTCTTTCAAGTT 47343501

Query 61901 AAGTTCTACATAATTTGAGGAATCATCATTTTGGCCTTTCAAGATAAATAAATCTGTTAC 61960

|||| |||||||||||||||||||||||||||||||||||||||||||||||||||||||

Sbjct 47343502 AAGTACTACATAATTTGAGGAATCATCATTTTGGCCTTTCAAGATAAATAAATCTGTTAC 47343561

Query 61961 TGTTC-CTC**G**TCTTCTTTGAACTCCACATGTCCATTTACTTTGAAGCACACAATAAGAAT 62019

|| || ||| |||||||||||||||||||||||||||||||||||||||||| |||||||

Sbjct 47343562 TG-TCTCTC**A**TCTTCTTTGAACTCCACATGTCCATTTACTTTGAAGCACACAGTAAGAAT 47343620

Query 62020 GACATTTCAAATTCTTGGAGCCAAAGGCTATGAAAATTATTTTGGCAGATTTTCTAAGAT 62079

| ||||||||||||||||||||||||||||||| ||||||||||||||||||||||||||

Sbjct 47343621 GGCATTTCAAATTCTTGGAGCCAAAGGCTATGACAATTATTTTGGCAGATTTTCTAAGAT 47343680

**rs2274700 (Position: 194,949,570; Exon 10 synonymous variant)**

**Query 194,949,497 – 194,949,675; Subject 195,120,368 - 195,120,545**

Query 62497 CCAAATCAAGTATAGATATTGAGAATGGGTTTATTTCTGAATCTCAGTATACATATGCCT 62556

||||||||||||| ||||||||||||||||||||||||||||||||||||| ||||| ||

Sbjct 47344099 CCAAATCAAGTATCGATATTGAGAATGGGTTTATTTCTGAATCTCAGTATATATATGACT 47344158

Query 62557 TAAAAGAAA-AAGC**G**AAATATCAATGCAAACTAGGATATGTAACAGCAGATGGTGAAACA 62615

|| | ||| |||| |||||||||||||||||||||||| ||||||||||||||||||||

Sbjct 47344159 TACAT-AAACAAGC**A**AAATATCAATGCAAACTAGGATATATAACAGCAGATGGTGAAACA 47344217

Query 62616 TCAGGATCAATTACATGTGGGAAAGATGGATGGTCAGCTCAACCCACGTGCATTAGTAAG 62675

|||||||||||||||||| |||| |||||||||||||||||||||| ||| |||||||

Sbjct 47344218 TCAGGATCAATTACATGTCAGAAAAATGGATGGTCAGCTCAACCCACATGCGTTAGTAA- 47344276

**rs10754199 (Position: 194,937,462; Intron 9)**

**Query 194,937,380 -194,937,555; Subject 195,103,761 - 195,103,938**

Query 50380 CGTAACTATTTGTGGCAAGCCAGGTCGAGGTCACTCTCTTTGAGGGCATTTACTCACTGC 50439

|||||||||||||||||||||||||||||| |||||| |||||||||||||||||| ||

Sbjct 47327492 TGTAACTATTTGTGGCAAGCCAGGTCGAGGTTACTCTCCTTGAGGGCATTTACTCACAGC 47327551

Query 50440 CTAGTGCCCCCTTTCCACTGGG**A**CAGACCCAGAGACAAACCCTAGCAGCTTACTTAGC- 50497

||||||||||||| ||||||| ||||||||||||||| | ||||||||| |||||

Sbjct 47327552 CTAGTGCCCCCTTGCCACTGGA**G**CAGACCCAGAGACAAGCACTAGCAGCTG-CTTAGTA 47327609

Query 50498 -T-CTCATGAACTAAGGGCTGTCCCTTAGTTCTCCCAAAGTAACCCACAATGCAACCCTG 50555

| || ||||||||| |||||||| ||||||||||| | |||||||||| || || ||

Sbjct 47327610 GTTCTGATGAACTAATGGCTGTCCTTTAGTTCTCCCCATGTAACCCACACTGTAATTTTG 47327669

**rs1048663 (Position: 194,941,605; Intron 9)**

**Query 194,941,554 - 194,941,672; Subject 195,107,604 - 195,107,722**

Query 54554 TTTGTTGGAACAGAAGTGGAGCTACTGACTTACCTCTAGAAGACTGAATAG**G**TATCAGGT 54613

||||| || |||||||||| ||||||||||| ||||||||||||||||| |||||||

Sbjct 47331335 TTTGTCAGAGCAGAAGTGGATCTACTGACTTAATTCTAGAAGACTGAATAG**A**TATCAGGC 47331394

Query 54614 CATCCT-CCTGGATAATCCTAGTGTTACTTTGAGAGCTGTTTCTGCTTTAAATCCTGCTA 54672

||| || |||| ||||||| ||||||||| |||||||||||||| || ||||||||||||

Sbjct 47331395 CAT-CTCCCTGCATAATCCCAGTGTTACTCTGAGAGCTGTTTCTACTCTAAATCCTGCTA 47331453

**rs1887973 (Position: 194,941,802: Intron 9)**

**Query 194,941,733 - 194,941,912; Subject 195,107,783 - 195,107,962**

Query 54733 ATGACCAAAATTCCCTTTAAAAACCTGGACTTAAAAATATTCACTGAGGAAAGCAGCTTT 54792

|||||||||||||||||||||||||||||||| ||||||||||||||||||||||||||

Sbjct 47331514 TTGACCAAAATTCCCTTTAAAAACCTGGACTTAGAAATATTCACTGAGGAAAGCAGCTTT 47331573

Query 54793 ATGCACCAC**G**GACAACAGAAGGCTGAGTATGCTATTGTAACCCTGTGACAGATCCTAGAT 54852

||| ||||| ||||||||||||||||||||||||| ||||||||||||||||||||||||

Sbjct 47331574 ATGAACCAC**A**GACAACAGAAGGCTGAGTATGCTATGGTAACCCTGTGACAGATCCTAGAT 47331633

Query 54853 GCAGAGACACTCCCTCTGGGTTCATCGGCACAGAAGGCAGAACTTAGAGTCCTAATCAGG 54912

|||||||||||| ||||||||||||| ||||| |||||||||||||||||||||| ||||

Sbjct 47331634 GCAGAGACACTCACTCTGGGTTCATCAGCACAAAAGGCAGAACTTAGAGTCCTAAACAGG 47331693

**rs402056 (Position: 194,938,609; Intron 9)**

**Query 194,938,542 - 194,938,719; Subject 195,105,163 - 195,105,339**

Query 51542 CTTGAACACTATCAAAACTGCATTTTGATCAGCCTCTGCAAGGGAGTGCCCAAGCAAAG- 51600

|| |||||||||||||||||||||||| ||||||||||||||||||||||||| ||||

Sbjct 47328894 CTCGAACACTATCAAAACTGCATTTTGGCCAGCCTCTGCAAGGGAGTGCCCAAGGAAAGC 47328953

Query 51601 GAGTCTCA**G**TAAGGTCCAGGATGGTCAAGCAGAAGCCTAATGAGGGTTGC-TTTGGAATT 51659

| | ||||||||||||||||| | |||||||||||||||||||| || || |||||||||

Sbjct 47328954 G-GCCTCA**G**TAAGGTCCAGGA-GATCAAGCAGAAGCCTAATGAGTGT-GCCTTTGGAATT 47329010

Query 51660 CTTAGAACAAGTCTTTGAAGCTTTCAGAGAATAAATGGATATTGACCCAGAAGCCCCAGA 51719

||||||||||||||||||||||||||| ||||||||||||||||| |||||||| ||||

Sbjct 47329011 TTTAGAACAAGTCTTTGAAGCTTTCAGACAATAAATGGATATTGACTCAGAAGCCTCAGA 47329070

**rs4658046 (Position: 194,937,380; Intron 9)**

**Query 194,937,320 - 194,937,497; Subject 195,103,701 - 195,103,878**

Query 50320 CCCCACTGTCTCAGTGTATTGTTCTGTTGCTGTGCAGCAGGCATACAAATCTGACAATCT 50379

|||||||||||||||||||||||||||||||||| |||||||||||||||||||| |||

Sbjct 47327432 TCCCACTGTCTCAGTGTATTGTTCTGTTGCTGTGCTGCAGGCATACAAATCTGACAGTCT 47327491

Query 50380 **C**GTAACTATTTGTGGCAAGCCAGGTCGAGGTCACTCTCTTTGAGGGCATTTACTCACTGC 50439

|||||||||||||||||||||||||||||| |||||| |||||||||||||||||| ||

Sbjct 47327492 **T**GTAACTATTTGTGGCAAGCCAGGTCGAGGTTACTCTCCTTGAGGGCATTTACTCACAGC 47327551

Query 50440 CTAGTGCCCCCTTTCCACTGG-GACAGACCCAGAGACAAACCCTAGCAGCTTACTTAGC- 50497

||||||||||||| ||||||| | ||||||||||||||| | ||||||||| |||||

Sbjct 47327552 CTAGTGCCCCCTTGCCACTGGAG-CAGACCCAGAGACAAGCACTAGCAGCTG-CTTAGTA 47327609

**rs10922102 (Position: ; Intron 9)**

**Query 194,934,826 - 194,935,002; Subject 195,101,145 - 195,101,321**

Query 47826 TTTTAAAAACCCATAGCAAATATCATACT-AA-ATATGTGTTCATTTTTATAAGAAATAG 47883

| | | ||||||||||| ||||||||||| || | |||||| |||||||||||||||||

Sbjct 47324876 TATGACAAACCCATAGCCAATATCATACTGAATGTGTGTGTTTATTTTTATAAGAAATAG 47324935

Query 47884 ACAAACTGTTTTCCTGAGCGATCATA**C**ATTGTACCTTCACATACTCAGTGTATGTGAGAT 47943

|||||||||||||||||| ||||||| ||| ||||||||||||||||||||||| ||| |

Sbjct 47324936 ACAAACTGTTTTCCTGAGTGATCATA**T**ATTCTACCTTCACATACTCAGTGTATGCGAGTT 47324995

Query 47944 CCAGTTGTTTC-GCAACCTTGCCAGCCTTTGAGATAATGTGTTTTTTTTTCCATTAAATT 48002

||| ||| ||| |||||| |||||||||||||| | ||| || |||||||||||| ||

Sbjct 47324996 CCAATTG-TTCTACAACCTCACCAGCCTTTGAGAT-A-GTGGTTGTTTTTCCATTAAGTT 47325052

**rs10922103 ({Position: 194,937,741; Intron 9)**

**Query 194,937,674 - 194,937,848; Subject 195,104,057 - 195,104,233**

Query 50674 GGCCACTGCATGGTTCTCATGCTTACAGTACGATCCTGTAGCTAGATTTGTTTTGTAAGA 50733

|||||||||||||||||||| |||||||||| |||||||||||||||||||| |||| ||

Sbjct 47327788 GGCCACTGCATGGTTCTCATACTTACAGTACCATCCTGTAGCTAGATTTGTTATGTATGA 47327847

Query 50734 AGGAAGAG**G**AATGAGATGAAATACCCT-ATGTATAATGTTTTATGTTGCTTTGGAAAAGT 50792

|||||||| |||| ||| |||| |||| |||||||||||||||||||||||||| |||||

Sbjct 47327848 AGGAAGAG**A**AATGGGATAAAAT-CCCTTATGTATAATGTTTTATGTTGCTTTGGTAAAGT 47327906

Query 50793 ACAACAATGTAGAAAAA--GGTAAAATCATGA-TAAAGTAAGAAATTAAAGAC-TGTTTG 50848

| | ||||||||||||| || ||||| |||| | | | ||||||||||| || ||||||

Sbjct 47327907 AGATCAATGTAGAAAAAACGGAAAAATTATGAATTA-GCAAGAAATTAAA-ACCTGTTTG 47327964

**rs4657826 (Position: 194,927,236; Intron 9)**

**Query 194,927,147 - 194,927,321; Subject 195,095,320 - 195,095,498**

Query 40147 ATATCAACAGTTACTTCTT---T-TTTTACATGTTAATGATTGGTGATGTCACATTTTAA 40202

|||||||||||| |||||| | ||||||||||||||||||||||||||||||||||||

Sbjct 47319051 ATATCAACAGTTCCTTCTTAGGTGTTTTACATGTTAATGATTGGTGATGTCACATTTTAA 47319110

Query 40203 TATTTACAAATTGAATGGCTATAAAATGTTATA**A**CATTATAGTGTAAATTTGCATTTCTT 40262

|||||||||||||| ||||||||||||||| || |||| ||||||||||||||||||| |

Sbjct 47319111 TATTTACAAATTGAGTGGCTATAAAATGTTTTA**C**CATTGTAGTGTAAATTTGCATTTCCT 47319170

Query 40263 TTACCACTAACTGGGTTTAGAATATATATTCACAGTTTAATTGGTATTTAAT-GCTTCTA 40321

||| ||||| ||||||| |||||||||||||||||||||||||||||| || | |||||

Sbjct 47319171 TTATTACTAATTGGGTTTGGAATATATATTCACAGTTTAATTGGTATTTGATTG-TTCTA 47319229

**rs10922096 (Position: 194,929,082; Intron 9)**

**Query 194,928,990 - 194,929,169 Subject 195,097,009 -195,097,184**

Query 41990 GTGTATCCTTTATGAATGCAAACTGGCTGTAGTTTCCTTTAAAATAGTCATTTAAATAAA 42049

|||| |||| |||| |||||||||||||| ||||||||||||||||||| ||||| |||

Sbjct 47320740 GTGTGTCCTGCATGAGTGCAAACTGGCTGTGGTTTCCTTTAAAATAGTCACTTAAAGAAA 47320799

Query 42050 AAATTTGCCAGATAAATCACAGAATATCAATT**T**CTCTTGACTTGTAAAACTTGAATTACT 42109

| |||||||| ||| |||||||||||||||||| ||| ||||||||||||||||| ||||

Sbjct 47320800 ACATTTGCCAAATACATCACAGAATATCAATT**T**TTCTGGACTTGTAAAACTTGAAATACT 47320859

Query 42110 AGTGCCATCTGAATGATTCTTCTGAAGATAGACAGAGTAGTCTCTACTTACCTGTGAGAG 42169

||||| |||||||||||||||||||| |||| ||||||| | |||| ||||| |||

Sbjct 47320860 GGTGCCTTCTGAATGATTCTTCTGAAGTTAGA----GTAGTCTGTCCTTATCTGTGGGAG 47320915

**rs1292471/rs529899 (Position: 194,934,051; Intron 9)**

**Query 194,933,977 - 194,934,147; Subject 195,100,267 - 195,100,443**

Query 46977 TAAGATCAGAGCAGACCT---GGAGATAGAGACA-TGAAAACCCTTCAAAAAATCAATGC 47032

||||| ||| |||||||| ||||||||||||| |||||||||| ||||||||||||

Sbjct 47323998 TAAGAACAGTGCAGACCTGAAGGAGATAGAGACACAAAAAACCCTTC-AAAAATCAATGC 47324056

Query 47033 ACCCAGGAGCTGGTTTTT**A**GAAAAGATT-A-ACAA-AG-TACACTACTAGCCATACTAAT 47088

| |||||||||||||||| | |||||| | |||| || || || ||||||||||| ||

Sbjct 47324057 ATCCAGGAGCTGGTTTTT**T**G--AAGATTAACACAATAGATAGACCACTAGCCATACCAAA 47324114

Query 47089 AAAGAAGAAAGGACAGAAGAATCAAATAGACACAAT-AAAAATGATAAAGAGGGTATCAC 47147

|||||| ||| || |||||||||||||||||||||| |||||||||||||||| ||||||

Sbjct 47324115 AAAGAAAAAAAGAGAGAAGAATCAAATAGACACAATAAAAAATGATAAAGAGGATATCAC 47324174

**rs203688 (Position: 194,939,008; Intron 9)**

**Query 194,938,899 - 194,939,078; Subject 195,105,520 - 195,105,698**

Query 51899 AATGGCAAGCTGACTTGTTATCTGTGGCTCTGACCCTAGTAGTCCCTGGACCACAACAAG 51958

| | |||||||||||||| |||||||||||||||||||||||||||||||||||||||

Sbjct 47329251 GA-GAAAAGCTGACTTGTTAGCTGTGGCTCTGACCCTAGTAGTCCCTGGACCACAACAAG 47329309

Query 51959 GGCCGTCATCAGACACTCCATCTATGGTAGAACCACCTGGGCCCCCAAA**G**GCCAATAAAA 52018

|||| |||||||| ||||||||| ||||| |||||||||||||||||| ||||| ||||

Sbjct 47329310 GGCCCTCATCAGATGCTCCATCTAAGGTAGGACCACCTGGGCCCCCAAA**A**GCCAAAAAAA 47329369

Query 52019 ACGGACATCCCATTGCAGGTCCCAACCAGTGTGCTTACTGCAACAGGGGGGACACTGAGT 52078

| ||||||||||||||||||||||| ||||||||||||||||||||||||||||||||

Sbjct 47329370 AGGGACATCCCATTGCAGGTCCCAAGCAGTGTGCTTACTGCAACAGGGGGGACACTGAAA 47329429

**rs1831282 (Position: 194,940,616; Intron 9)**

**Query 194,940,544 - 194,940,723; Subject 195,106,596 - 195,106,775**

Query 53544 ACTGAGGATTATTGGTTTGTACAGGATTTGAGGGCTACTAGGCAAATTTTCAAAGCCATT 53603

||| |||| ||||| || |||||||||||||||||||||| ||||||||||||| ||||

Sbjct 47330327 ACTCAGGAATATTGATTCATACAGGATTTGAGGGCTACTAGCCAAATTTTCAAAGTCATT 47330386

Query 53604 TATCTGGTGATA**A**CTGATGCTTATACATTATTCATGACTTTAACCAGTGAGTTGTACTGG 53663

||||||||| || | |||||||||||||||||||||||||||||||||||||||||||||

Sbjct 47330387 TATCTGGTGGTA**C**CCGATGCTTATACATTATTCATGACTTTAACCAGTGAGTTGTACTGG 47330446

Query 53664 TGTTCAGTCTTGAATCTGAAGGATGCCTTCATCTGTATTCCCCTGAGTCCAGAGTCCCAT 53723

|||||||||||| || |||| ||||||||||| |||||||| ||||||||||||||||||

Sbjct 47330447 TGTTCAGTCTTGGATTTGAAAGATGCCTTCATTTGTATTCCTCTGAGTCCAGAGTCCCAT 47330506

**rs2019727 (Position: 194,941,337; Intron 9)**

**Query 194,941,257 - 194,941,434; Subject 195,107,305 - 195,107,483**

Query 54257 TTTCTAACCATTAAGTAAAAATTACTAATGGATCTGGCTACTGAG-ACCCCCTGAACTAA 54315

|||||||||||||| |||||||| |||||| ||||||||||| | ||||||||||||||

Sbjct 47331036 TTTCTAACCATTAAATAAAAATTGTTAATGGCTCTGGCTACTG-GTACCCCCTGAACTAA 47331094

Query 54316 GAAAGCCATTTGATTTGTTCA**T**ACATGAGAGAGAAGGGATGGGTTTAGGAGTAGTAACCC 54375

||||||||||| ||||||||||| |||||||||||||| || ||||||||||| ||||||

Sbjct 47331095 GAAAGCCATTTAATTTGTTCA**T**ATATGAGAGAGAAGGGGTGAGTTTAGGAGTACTAACCC 47331154

Query 54376 AAGACTTGGGGAATATCATGAGGCCTGTAGCCTACTTTTCAAAACAGCTGGACATTGT-T 54434

||||||||||||||||||||||||||||||||||||||||||||||||||||||||||

Sbjct 47331155 AAGACTTGGGGAATATCATGAGGCCTGTAGCCTACTTTTCAAAACAGCTGGACATTGTCC 47331214

**rs2019724 (Position: 194,941,540; Intron 9)**

**Query 194,941,435 - 194,941,613; Subject 195,107,484 - 195,107,663**

Query 54435 ATGA-TGGGTTGGACTTCTTATCTCTGAGCCACTGGCACCACTTGTGATCTTCTCCAGGA 54493

| | ||||||||||||||| |||||||||| ||| |||||||||||||||||||||||

Sbjct 47331215 AAAACTGGGTTGGACTTCTTGCCTCTGAGCCATTGGTACCACTTGTGATCTTCTCCAGGA 47331274

Query 54494 GGCAGAAGAGTTCACTTTGGGTTAACCTAGCACTGTACACACCCCA**T**ACTGTGTATGCTA 54553

||||||||||||||||||||||||||||||||| |||||||||||| ||| ||||||||

Sbjct 47331275 GGCAGAAGAGTTCACTTTGGGTTAACCTAGCACAGTACACACCCCA**C**ACTATGTATGCTC 47331334

Query 54554 TTTGTTGGAACAGAAGTGGAGCTACTGACTTACCTCTAGAAGACTGAATAGGTATCAGGT 54613

||||| || |||||||||| ||||||||||| ||||||||||||||||| |||||||

Sbjct 47331335 TTTGTCAGAGCAGAAGTGGATCTACTGACTTAATTCTAGAAGACTGAATAGATATCAGGC 47331394

**rs6428357 (Position: 194,942,194; Intron 9)**

**Query 194,942,090 - 194,942,268; Subject 195,108,135 - 195,108,309**

Query 55090 TTGGTACCCAAGAAAGTAGCTATAATACACTACCATGGACATCAGTGGATGGACAATTTG 55149

|||||||||||||||||||||||| ||||| ||| |||||||||||||||||||||||||

Sbjct 47331866 TTGGTACCCAAGAAAGTAGCTATACTACACCACCCTGGACATCAGTGGATGGACAATTTG 47331925

Query 55150 GTAGCAAGAAAAAAAAAATCACTAGGCAGAGCAGGCCACTAAAG**A**GGTGAACAGAGAAA 55208

|||||||| ||||||||||||||||||| ||||||||||||| | ||| ||||| ||

Sbjct 47331926 GTAGCAAG---AAAAAAATCACTAGGCAGACCAGGCCACTAAAG**G**GATGACCAGAG-AA 47331980

Query 55209 AAAATGCCCAAAGCCTTCCTAATGCCATTAATCCCTGAAATAAACTTCAGCCTAAAATCC 55268

||| | ||||||||||| |||||||||||||||||||||||||||||||||||||||||

Sbjct 47331981 AAAGTACCCAAAGCCTTGCTAATGCCATTAATCCCTGAAATAAACTTCAGCCTAAAATCT 47332040

**rs6664705 (Position: 194,944,465; Intron 9)**

**Query 194,944,356 - 194,944,535; Subject 195,110,362 - 195,110,541**

Query 57356 TATTCCCCATGAAAAAATCCATCAATAAATGCACCCTTGAGGGCTTTACCTGTGTGCCCC 57415

||||||||||||||||| ||||||||| ||||| | |||||||||| || |||||| |||

Sbjct 47334093 TATTCCCCATGAAAAAAACCATCAATACATGCATCTTTGAGGGCTTCACTTGTGTGTCCC 47334152

Query 57416 CTTGGTACATGTTTATTTGTGGCATAGGATCAGACCCTCCTCTGTTAGG**G**CAAGCCCACT 57475

||||| | ||||||||||||| || ||||||||| | |||||||||||| ||||| ||||

Sbjct 47334153 CTTGGCAAATGTTTATTTGTGACACAGGATCAGAACATCCTCTGTTAGG**A**CAAGCTCACT 47334212

Query 57476 GGTGTCTCAATAACCTGTATATTAAGGGCTCTCACTTACTGAGATACTTCTCAGTCCATC 57535

||||||||||||||||||||||| |||||||| ||||| || || ||| ||||||||||

Sbjct 47334213 GGTGTCTCAATAACCTGTATATTGAGGGCTCTTACTTATTGGGACACTGGTCAGTCCATC 47334272

**rs203685 (Position: 194,944,568; Intron 9)**

**Query 194,944,536 - 194,944,654; Subject 195,110,542 - 195,110,661**

Query 57536 CTAATGACAAAATATTCTGCCCATTTTCCCAC**C**GACTCCAAAAGGGACTGCCAGGCATAT 57595

|||||||| ||| ||||||||||||||||||| ||||||||||| ||||||||||||||

Sbjct 47334273 CTAATGACTAAAGATTCTGCCCATTTTCCCAC**G**GACTCCAAAAGCCACTGCCAGGCATAT 47334332

Query 57596 ATCAATATATCTGGTGGCAGAGCCTTC-TAGGCATCACGAGTCCTAGCTATGGAGTATAC 57654

||||||||||| |||| ||| |||||| | |||||||| | ||||||||||| |||||||

Sbjct 47334333 ATCAATATATCCGGTGACAGGGCCTTCTTGGGCATCACAATTCCTAGCTATGAAGTATAC 47334392

**rs203682 and rs10737679 (Position: 194,945,818 and 194,945,859; Intron 9)**

**Query 194,945,752 - 194,945,924; Subject 195,111,794 - 195,111,965**

Query 58752 ATCATCTTGCTCAAAAACAAAACACACACACACACACAAACATACACACACAGACACACA 58811

| ||| | ||||||||||||| | ||| | | | ||| ||| ||||||||| |||||||

Sbjct 47335525 ACCATGT--CTCAAAAACAAAA-A-ACA-A-ATATACATACACACACACACACACACACA 47335578

Query 58812 GACACA**C**ACATCAGCTGGTCTAAAGAGCCCAGCAAAAAGCTGACTC**A**GGAAATAATGCAG 58871

||||||| ||||||||||||||||||||||||||||||||||||||||||||||||||

Sbjct 47335579 TACACA**C**A--TCAGCTGGTCTAAAGAGCCCAGCAAAAAGCTGACTC**A**GGAAATAATGCAG 47335636

Query 58872 GTTCCACATCC----T---GGTGACTTCATCCCCCTTACCCTGACCAATTGACAACCCAA 58924

||||||||||| | ||||| |||||||||||||||||||||||||||||||||||

Sbjct 47335637 GTTCCACATCCCACATCCCGGTGATTTCATCCCCCTTACCCTGACCAATTGACAACCCAA 47335696

**rs10737680 (Position: 194,946,078; Intron 9)**

**Query 194,945,985 - 194,946,164; Subject 195,112,026 - 195,112,205**

Query 58985 AGAACAGATCTGGGGCTTGAGAATCCTCCCAATTCCTTGTTTGGTGCCCTTGTGTTGATT 59044

||||||||| ||||||||||||||||||||| |||||||||||||||||||| ||||||

Sbjct 47335757 AGAACAGATTTGGGGCTTGAGAATCCTCCCATTTCCTTGTTTGGTGCCCTTGCATTGATT 47335816

Query 59045 AAAGCCTTTCTTTGCTGCAAACCCTACTGTCTC**A**GCGTATTGGTCTATTGCTAAACAGTG 59104

|||| |||||||||||||||| |||||||||||| ||||||||||||||||| ||||||

Sbjct 47335817 AAAGTTTTTCTTTGCTGCAAACTCTACTGTCTC**A**GTGTATTGGTCTATTGCTAGACAGTG 47335876

Query 59105 GGCATGTGAACCTGATAGTCTTATAACAAAATAGAATAGAAGCTATTTATAGGATCGATC 59164

||||||||||||||||||| ||||||||||||||||||||||||||||| | |||||||

Sbjct 47335877 GGCATGTGAACCTGATAGTGTTATAACAAAATAGAATAGAAGCTATTTACAAAATCGATC 47335936

**rs1831281 (Position: 194,947,437; Intron 9)**

**Query 194,947,398 - 194,947,517; Subject 195,118,776 - 195,118,894**

Query 60398 AAACCCCAGAGATAGTTTGTTCAGAATATTAAGCTGCTT**C**TGATGTCTCTACTACGTGAC 60457

||||| |||||||||||||||||||||||||||||||||||||||||||||||| ||||

Sbjct 47342507 AAACCGCAGAGATAGTTTGTTCAGAATATTAAGCTGCTT**C**TGATGTCTCTACTAAATGAC 47342566

Query 60458 CTAGATGTTCAAACGAGTCTTTCTACTCTGATTGATTCCATTTAAATGTCTCCCTGTGTT 60517

|||||||||||||||||||||||||||| ||||||||||||||||||||||||||||| |

Sbjct 47342567 CTAGATGTTCAAACGAGTCTTTCTACTCCGATTGATTCCATTTAAATGTCTCCCTGTG-T 47342625

**rs203679 (Position: 194,949,141; Intron 9)**

**Query 194,949,080 - 194,949,258; Subject 195,119,950 - 195,120,129**

Query 62080 CATCACAGGATCTCAGATACTCCAATTGGCTAAATGAGATTTGGAATTGCC-AGAAGATC 62138

|||||| |||||||||| ||||||||||| |||||||| |||||||||||| | ||||||

Sbjct 47343681 CATCACGGGATCTCAGACACTCCAATTGGTTAAATGAGTTTTGGAATTGCCTAAAAGATC 47343740

Query 62139 TA**C**AATAGGCAAGATTAAAAAGTCCTAAGAAATAATACTCATTGTCTATTGAGTTCTGAC 62198

|| ||||||||||||||||||||| |||||||||||||||||||||||||||||||||||

Sbjct 47343741 TA**A**AATAGGCAAGATTAAAAAGTCTTAAGAAATAATACTCATTGTCTATTGAGTTCTGAC 47343800

Query 62199 ATGCTGTAGAATTCCAAATCCATTAGACAAACAGATTTCCAATAATTTCTTAGAAATAAT 62258

|||||||||||||||||||||||||||| |||||||||||||||||||||||||||||||

Sbjct 47343801 ATGCTGTAGAATTCCAAATCCATTAGACCAACAGATTTCCAATAATTTCTTAGAAATAAT 47343860

rs2860102 (Position: 194,934,942; Intron 9)

**Query 194,934,826 - 194,935,002; Subject 195,101,145 - 195,101,321**

Query 47826 TTTTAAAAACCCATAGCAAATATCATACT-AA-ATATGTGTTCATTTTTATAAGAAATAG 47883

| | | ||||||||||| ||||||||||| || | |||||| |||||||||||||||||

Sbjct 47324876 TATGACAAACCCATAGCCAATATCATACTGAATGTGTGTGTTTATTTTTATAAGAAATAG 47324935

Query 47884 ACAAACTGTTTTCCTGAGCGATCATACATTGTACCTTCACATACTCAGTGTATGTGAG**A**T 47943

|||||||||||||||||| ||||||| ||| ||||||||||||||||||||||| ||| |

Sbjct 47324936 ACAAACTGTTTTCCTGAGTGATCATATATTCTACCTTCACATACTCAGTGTATGCGAG**T**T 47324995

Query 47944 CCAGTTGTTTC-GCAACCTTGCCAGCCTTTGAGATAATGTGTTTTTTTTTCCATTAAATT 48002

||| ||| ||| |||||| |||||||||||||| | ||| || |||||||||||| ||

Sbjct 47324996 CCAATTG-TTCTACAACCTCACCAGCCTTTGAGAT-A-GTGGTTGTTTTTCCATTAAGTT 47325052

**rs10801555 (194,926,884: Intron 9)**

**Query 194,926,795 - 194,926,971; Subject 195,094,964 - 195,095,139**

Query 39795 T-GTTAGAAAA-GAAAATGATTTGGGGGCTGCCT-TTCATTAAAAGGAAAAACCTTATCA 39851

| ||||||||| |||| ||||||||||||| ||| || ||||||||||||| | ||| ||

Sbjct 47318695 TTGTTAGAAAAAGAAA-TGATTTGGGGGCT-CCTATT-ATTAAAAGGAAAA-CTTTACCA 47318750

Query 39852 AGGACTGCTGTACCCTCATTATCTGCCTAAA**C**AATTTTTTCTCAACTCCTATATCAATAT 39911

|||||| |||||||||||||||||||||||| ||||| |||| ||||| | |||||||||

Sbjct 47318751 AGGACTCCTGTACCCTCATTATCTGCCTAAA**T**AATTTCTTCTTAACTCGTGTATCAATAT 47318810

Query 39912 TAATATGTACATTGGAATCAATCATTTGACTCAAAATAATTATACAATTATAGTTTAAAA 39971

|||||||||| |||||||||||||||||||||||||| |||||||| |||||||||||||

Sbjct 47318811 TAATATGTACTTTGGAATCAATCATTTGACTCAAAATGATTATACATTTATAGTTTAAAA 47318870
