# Supplementary material for: A 32 kb Critical Region Excluding Y402H in CFH Mediates Risk for Age-Related Macular Degeneration
Source: PLoS One. 2011 Oct 12;6(10):e25598. doi: 10.1371/journal.pone.0025598 (PMC3192039; doi:10.1371/journal.pone.0025598)
Supplement: Text S2 — Supplementary methods. (DOC) [file pone.0025598.s021.doc]

**Text S2. Supplementary Methods**

***FARMS cohort***

The FARMS set consists of 34 index cases with advanced AMD and their family members, who visited the Retinal Clinic at the University of Wisconsin. All subjects (a total of 297 individuals and 349 sib pairs) who consented were interviewed and had fundus photographs taken. Family members who lived at remote sites were sent to the local clinic for blood draws and photographs. The protocol for phlebotomy and subject testing was approved by the Institutional Review Board at the University of Wisconsin. Fundus photographs were graded using a codified grading scheme that is described elsewhere [2,3]. A 16-level (0–15) severity scale, called Wisconsin Age-Related Maculopathy Grading system (WARMGS) score, was constructed based on drusen size, type, and area, pigmentary abnormalities, geographic atrophy, and signs of exudative macular degeneration. The ordering of the scale was based on associations of the presence and absence of early signs of age-related maculopathy with the incidence of geographic atrophy or exudative macular degeneration. Cases were identified as subjects with WARMGS score greater than 11 in either eye at baseline or follow up visit. A WARMGS score of 11 or above corresponds to classification as a category 3 (WARMGS 11), 4 or 5 (WARMGS12-15) on the AREDS scale, described below. Subjects classified as having WARMGS score of 4 or less correspond to AREDS category 1.

***AREDS cohort***

AREDS is a long-term multicenter prospective study that was designed with the primary support of the National Eye Institute (NEI) to assess the clinical course of, and risk factors for, the development and progression of age related cataract and AMD. Starting in November 1992, the study enrolled 4757 participants, who were between 55 and 80 years of age and are free of any illness or conditions that would make long-term follow-up or compliance with study medications unlikely or difficult. The data on possible risk factors, changes in visual acuity, photographic documention of changes in macula or lens status, and self-reported visual function were collected from all the participants. Eyes that developed the neovascular form of the disease or geographic atropy were considered to have advanced AMD. Other lesions of AMD, such as drusen (size, type and extent) and Retinal Pigment Epithelium abnormalities (detachment, atropy and pigment disturbances) are individually graded for each study eye. Based on these grading all the participants were divided into five groups according to the size and extent of drusen in the eye, presence of geographic atrophy (GA), and choroidal neovascular (CNV or NV_AMD) changes and are described in AREDS report 3 [4]. To avoid confusion between copy number variations (CNV) and choroidal neovascular (CNV or NV_AMD), we will refer to choroidal neovascular type of AMD as NV_AMD in the manuscript. Briefly, group 1 was classified as controls and groups 2 and 3 were defined as intermediate and large drusen, respectively. Cases with GA were classified as group 4 and NV cases are assigned to be group 5.

A total of 600 individuals (400 AMD cases and 200 controls) with clearly defined phenotypes were selected for the genome-wide association study. These individuals were categorized into six different groups based on the AMD disease status: (1) 138 cases who had GA either central or non-central to the macula at their most recent study visit, (2) 198 individuals with NV_AMD, (3) 50 participants with both GA and CNV or NV_AMD, (4) 14 cases with Large Drusen (>125 um in diameter), (5) 72 individuals who had few or no drusen (< 63 μm in diameter) during the study period and (6) 28 controls who developed moderate disease during the course of the study (assigned as controls questionable).

***Michigan-Penn-Mayo Panel***

This panel includes the 3307 (2157 cases and 1150 controls) samples collected from University of Michigan in  Ann Arbor, the University of Pennsylvania in Philadelphia, Mayo Clinic in Rochester and AREDS. All samples were evaluated by an Ophthalmologist and/or had fundus grading done by either a retina specialist or a retinal grading center. Genotyping was performed at the Center for Inherited Disease Research (CIDR) at Johns Hopkins University using Illumina Human370 Bead Chips and the Illumina Infinium II assay protocol. A total of 238 AREDS samples that were included in the custom illumina panel as well as in AREDS GWAS study were excluded from further analysis.

***Indian case-control cohort***

The case-control population consisted of 251 individuals of south Indian ethinicity, aged more than 50 years of age of which there were 134 unrelated cases and 117 age-matched controls. These subjects were recruited from the Vitreo-Retinal outpatient clinic at Sankara Nethrayala, Chennai, India. The ancestral origin/ethnicity of patients was established through self-reporting or an open questionnaire.

All subjects underwent a complete ophthalmic examination including recording of the best-corrected visual acuity, subjective refraction, slit lamp examination, applanation tonometry, binocular indirect ophthalmoscopy and stereoscopic evaluation of disc and macula using the +78 D lens. A 30-degree macula-centered color photograph was taken for each eye after pharmacologic mydriasis with tropicamide 0.5% and phenylephrine 5%. The Zeiss FF450-plus fundus camera with VISUPAC digital image archiving system (Carl Zeiss, Jena, Germany) was used. Evaluation of the fundus photographs was done using the VISUPAC digital image archiving system. All images were screened and graded by a retinal specialist for presence / absence and classification of ARM. Age related maculopathy was classified using the modified grading system used in the Age related Eye Disease Study. Control subjects were recruited through the ongoing Chennai Glaucoma epidemiology study [5]. Control subjects did not have any sign of ARM, in form of drusen or RPE abnormalities. Subjects with stage 4 disease in one or both the eyes were defined to have late ARM. More than 80% of cases presented with late-stage disease.

***Singapore Malay Eye Study (SiMES)***

SiMES is a population-based cross-sectional study of Malay adults aged 40 to 80 years living in Singapore. The study design and details of the population sampling of the SiMES have been described elsewhere [6–8]. All the 3280 participants underwent fundus photography, which were graded for presence or absence of AMD using WARMGS score [3,9]. A total of 3072 individuals were genotyped with Illumina Human 610K bead array. After all the quality control procedure, 140 cases and 2390 controls were included in this study.

***Custom Illumina Panel***

*BDES Cohort:* Two hundred seventy-seven cases and 764 controls were selected from a population based BDES cohort [10]. The BDES used a private census to identify individuals living in Beaver Dam, WI between the ages of 43 and 86 years to participate in a longitudinal study of ocular diseases of aging. A portion of the collected BDES cohort consisted of related individuals. For this current study, only one individual per family was selected to be used either as a case or a control, with cases given priority in selection. All participants underwent clinical exam, fundus photography and blood draw. Five, ten, and fifteen year follow-up exams with fundus photography have been conducted. Fundus photographs were scored using the WARMGS score. Based on this score, individuals at the extremes of the scale were selected as cases and controls, as described in the FARMS set. Controls were selected to be over age 60, to have been seen at all four exam periods, and to have not scored greater than a WARMGS score of 4 in either eye at any time point. This population consists of subjects of primarily Western European descent.

*CCF Cohort:* A total of 656 cases and 342 controls, who visited Cole Eye Institute at the Cleveland Clinic Foundation in Cleveland, OH, were included in this study. AMD status was determined by an ophthalmologist using a dilated visual exam. At the time of the exam, subjects were assigned to one of the AREDS categories. For the current study, participants were included as cases if they were categorized as being in AREDS categories 3 or 4. Controls also underwent clinical exam, were at least 60 years of age, and were free from any additional ocular disorders that could obscure the AMD phenotype (e.g. diabetic retinopathy). All included cases and controls were self-described as being white, of non-Hispanic origin.

*OHSU Cohort:* Participants were part of a sporadic case/control cohort identified at the Casey Eye Institute, Oregon Health and Science University, Portland, OR. All subjects underwent clinical exam and a blood draw. These individuals were categorized according to the AREDS grading scale: cases were defined as having advanced AMD (geographic atrophy or choroidal neovascularization) corresponding to AREDS category 4. Controls were free from AMD upon ophthalmologic exam (no drusen greater than 63 microns in diameter) and at least 60 years in age. All subjects (410 cases and 221 controls) were of Northern European Caucasian descent.

*VAMC Cohort:* Participants from this cohort were recruited at the Eye Clinic of Louis Stokes Cleveland Department of Veterans Affairs Medical Center, Cleveland, OH. All participants were evaluated similarly to the CCF cohort, with dilated visual exam, visual acuity testing and a blood draw. Subjects were categorized according to the AREDS criteria by the examining physician at the time of exam. Cases were defined as individuals with an AREDS categorization of 3 or 4, and controls were defined as subjects over age 60 with a normal fundus appearance on clinical exam, no visual abnormalities or family history of retinal disease. Although this cohort contains participants from other ethnicities (African Americans, Asians, etc.), only individuals of white, non-Hispanic origin were selected for this study. In total, 120 cases and 243 controls from this cohort were included for genotyping with a custom Illumina assay.

*AREDS Cohort:* An additional 478 cases and 99 controls were selected from the remaining AREDS participants. Samples on whom a genome wide association study was already performed were not genotyped using our custom Illumina panel. Cases and controls were chosen in a fashion similar to the subjects identified for use in the whole genome scan. Cases consisted of individuals with late stage AMD and the controls were free from AMD in either eye and over 65 years in age.

References

1. Thompson CL, Klein BEK, Klein R, Xu ZY, Capriotti J, et al. (2007) Complement factor H and hemicentin-1 in age-related macular degeneration and renal phenotypes. Human Molecular Genetics 16: 2135-2148.

2. Klein R (1991) Age-Related Eye Disease, Visual Impairment, and Driving in the Elderly. Human Factors 33: 521-525.

3. Klein R, Davis MD, Magli YL, Segal P, Klein BEK, et al. (1991) The Wisconsin Age-Related Maculopathy Grading System. Ophthalmology 98: 1128-1134.

4. Anand R, Bressler SB, Davis MD, Ferris FL, Klein R, et al. (2000) Risk factors associated with age-related macular degeneration - A case-control study in the Age-Related Eye Disease Study: Age-Related Eye Disease Study report number 3. Ophthalmology 107: 2224-2232.

5. Vijaya L, George R, Baskaran M, Arvind H, Raju P, et al. (2008) Prevalence of primary open,angle glaucoma in an urban south Indian population and comparison with a rural population: The Chennai Glaucoma Study. Ophthalmology 115: 648-654.

6. Su DHW, Wong TY, Wong WL, Saw SM, Tan DTH, et al. (2008) Diabetes, hyperglycemia, and central corneal thickness - The Singapore Malay Eye Study. Ophthalmology 115: 964-968.

7. Foong AWP, Saw SM, Loo JL, Shen S, Loon SC, et al. (2007) Rationale and methodology for a population-based study of eye diseases in Malay people: The Singapore Malay eye study (SiMES). Ophthalmic Epidemiology 14: 25-35.

8. Kawasaki R, Wang JJ, Aung T, Tan DTH, Mitchell P, et al. (2008) Prevalence of age-related macular degeneration in a Malay population - The Singapore Malay Eye Study. Ophthalmology 115: 1735-1741.

9. Klein R, Klein BEK, Linton KLP, Demets DL (1991) The Beaver Dam Eye Study - Visual-Acuity. Ophthalmology 98: 1310-1315.

10. Klein R, Klein BEK, Linton KLP (1992) Prevalence of Age-Related Maculopathy - the Beaver Dam Eye Study. Ophthalmology 99: 933-943.
