# Supplementary material for: A 32 kb Critical Region Excluding Y402H in CFH Mediates Risk for Age-Related Macular Degeneration
Source: PLoS One. 2011 Oct 12;6(10):e25598. doi: 10.1371/journal.pone.0025598 (PMC3192039; doi:10.1371/journal.pone.0025598)
Supplement: Supplementary file 12 [file pone.0025598.s004.doc]

CEU

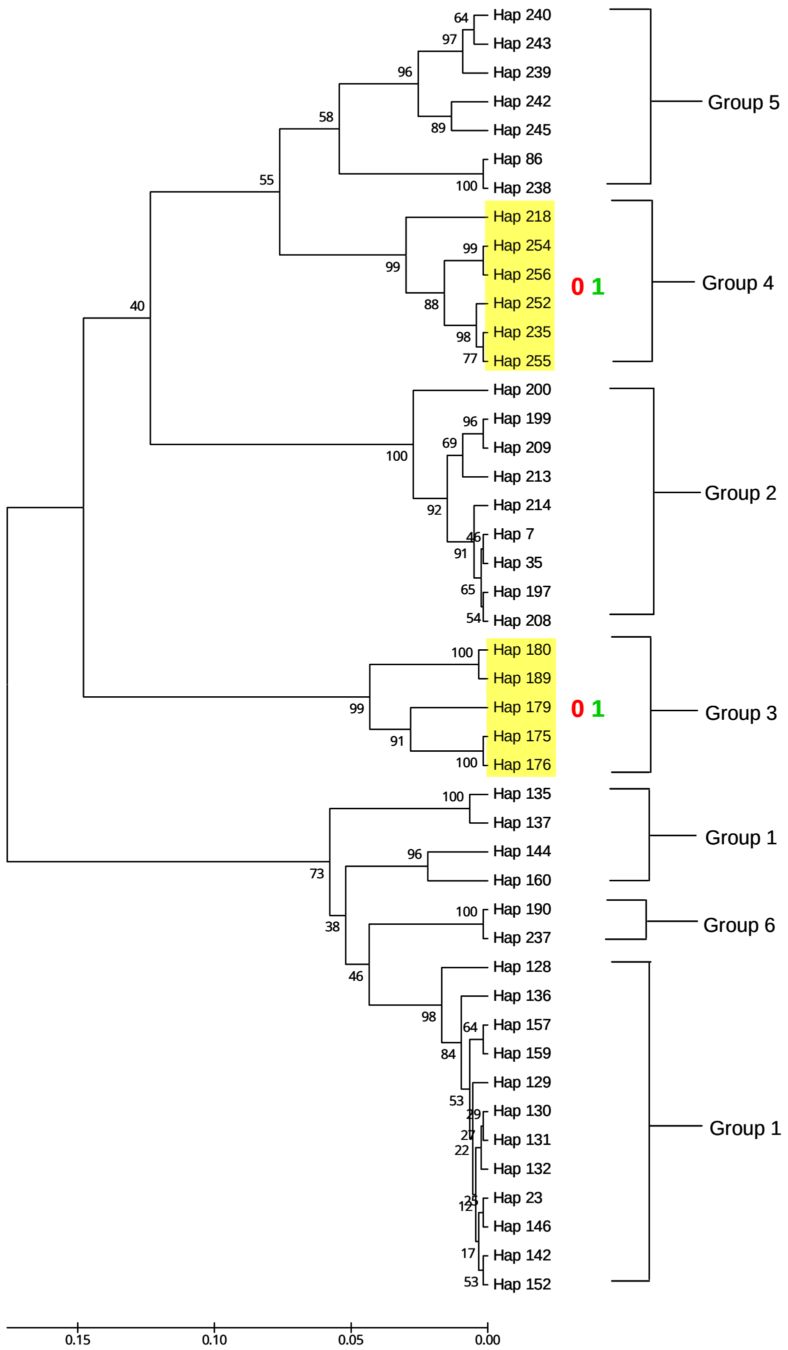

Hap 240
Hap 243
Hap 239
Hap 242
Hap 245
Hap 86
Hap 238
Group 5
Hap 218
Hap 254
Hap 256
Hap 252
Hap 235
Hap 255
0 1
Group 4
Hap 200
Hap 199
Hap 209
Hap 213
Hap 214
Hap 7
Hap 35
Hap 197
Hap 208
Group 2
Hap 180
Hap 189
Hap 179
Hap 175
Hap 176
0 1
Group 3
Hap 135
Hap 137
Hap 144
Hap 160
Group 1
Hap 190
Hap 237
Group 6
Hap 128
Hap 136
Hap 157
Hap 159
Hap 129
Hap 130
Hap 131
Hap 132
Hap 23
Hap 146
Hap 142
Hap 152
Group 1
0.15
0.10
0.05
0.00

CHB + JPT

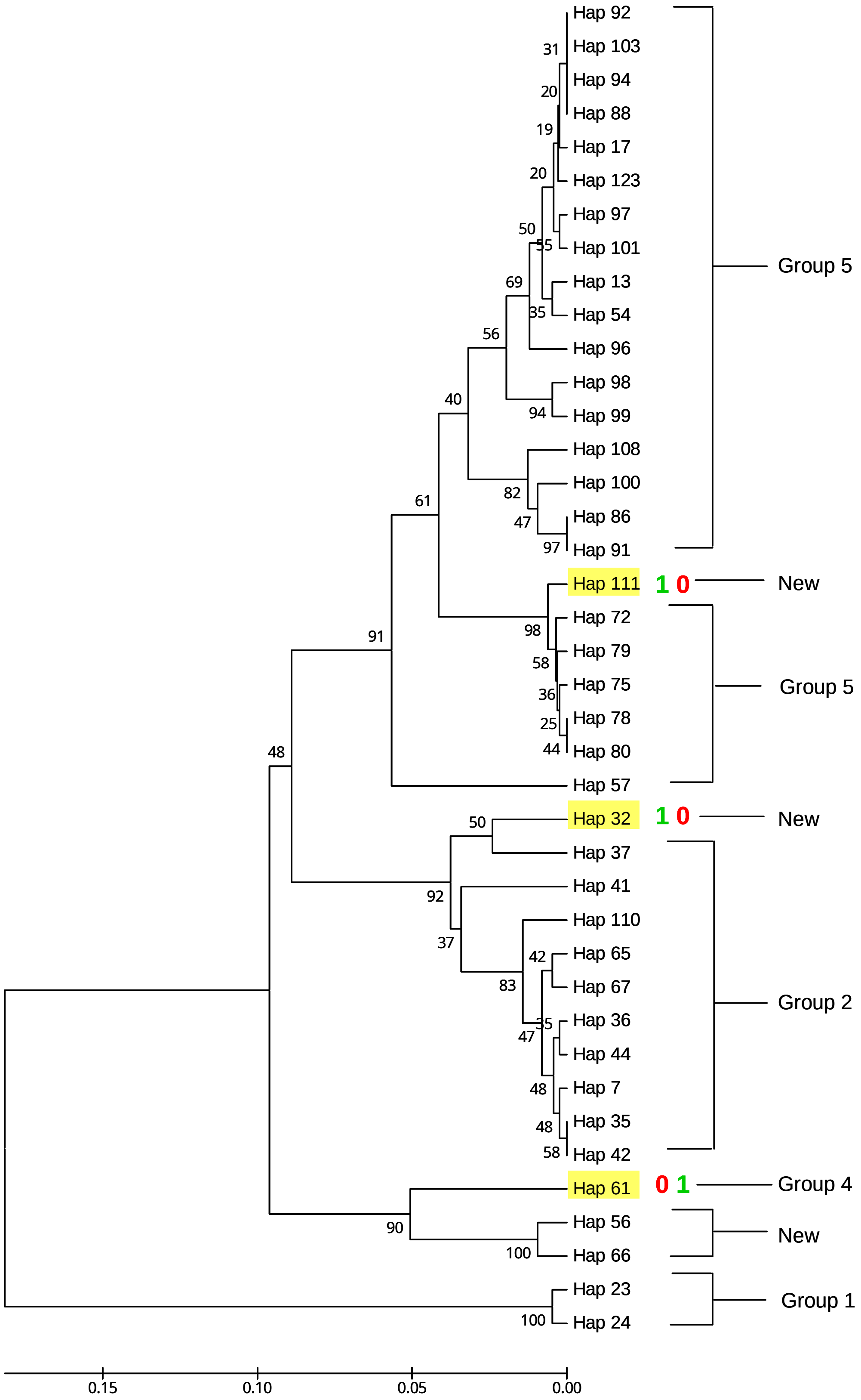

Hap 92
Hap 103
Hap 94
Hap 88
Hap 17
Hap 123
Hap 97
Hap 101
Hap 13
Hap 54
Hap 96
Hap 98
Hap 99
Hap 108
Hap 100
Hap 86
Hap 91
Group 5
Hap 111
1 0
New
Hap 72
Hap 79
Hap 75
Hap 78
Hap 80
Hap 57
Group 5
Hap 32
1 0
New
Hap 37
Hap 41
Hap 110
Hap 65
Hap 67
Hap 36
Hap 44
Hap 7
Hap 35
Hap 42
Group 2
Hap 61
0 1
Group 4
Hap 56
Hap 66
New
Hap 23
Hap 24
Group 1
0.15
0.10
0.05
0.00

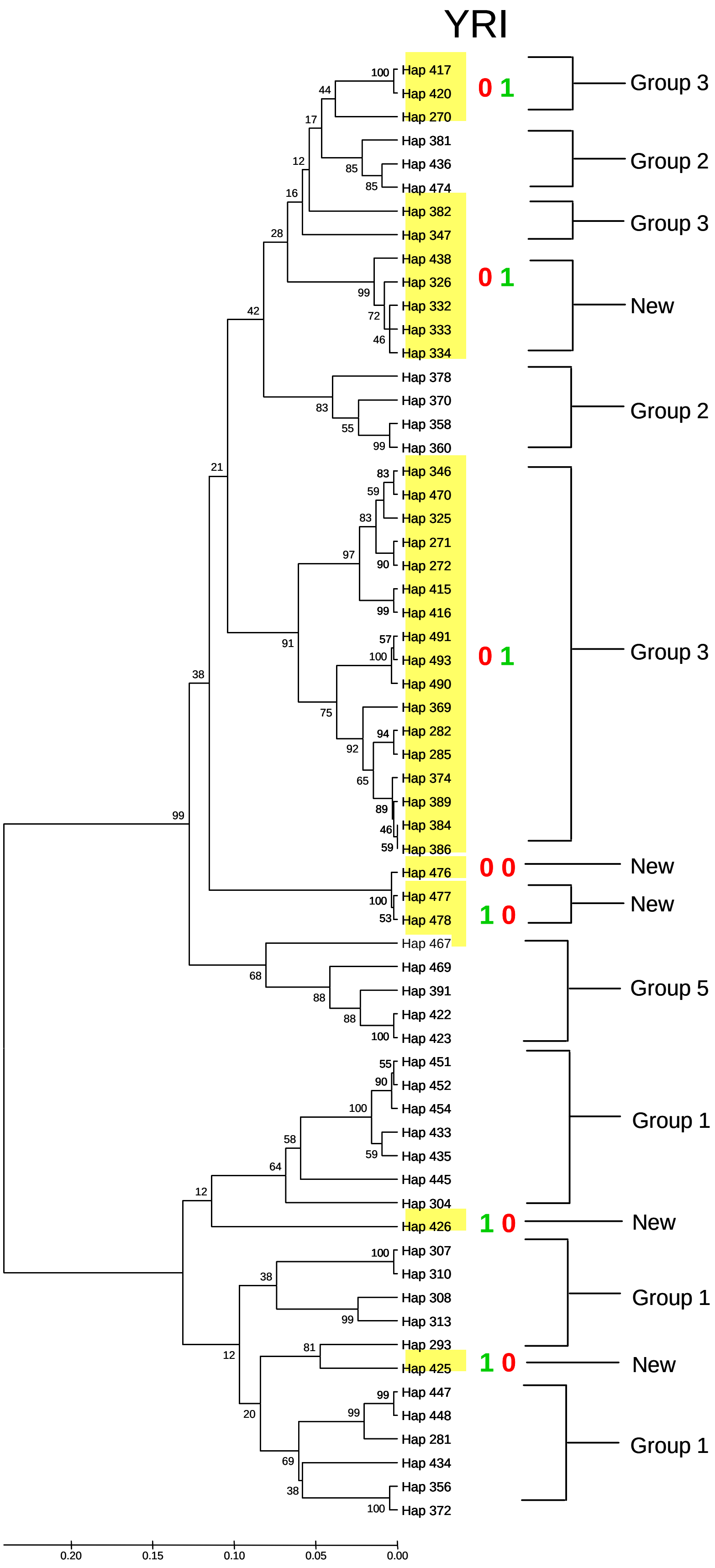

YRI
Hap 417
Hap 420
Hap 270
Hap 381
Hap 436
Hap 474
Hap 382
Hap 347
Hap 438
Hap 326
Hap 332
Hap 333
Hap 334
Hap 378
Hap 370
Hap 358
Hap 360
Hap 346
Hap 470
Hap 325
Hap 271
Hap 272
Hap 415
Hap 416
Hap 491
Hap 493
Hap 490
Hap 369
Hap 282
Hap 285
Hap 374
Hap 389
Hap 384
Hap 386
Hap 476
Hap 477
Hap 478
Hap 467
Hap 469
Hap 391
Hap 422
Hap 423
Hap 451
Hap 452
Hap 454
Hap 433
Hap 435
Hap 445
Hap 304
Hap 426
Hap 307
Hap 310
Hap 308
Hap 313
Hap 293
Hap 425
Hap 447
Hap 448
Hap 281
Hap 434
Hap 356
Hap 372
0 1
0 1
0 1
0 0
1 0
1 0
1 0
Group 3
Group 2
Group 3
New
Group 2
Group 3
New
New
Group 5
Group 1
New
Group 1
New
Group 1
0.20
0.15
0.10
0.05
0.00
